# Integrin activation by two independently regulated calcium-mediated pathways is required for neutrophil recruitment

**DOI:** 10.1186/s12964-026-02666-w

**Published:** 2026-01-21

**Authors:** Marina Oguama, Pia Lindental, Regina A. Clemens, Clifford A. Lowell, Oliver Soehnlein, Johannes Roth, Tamam Bakchoul, Anika Cappenberg, Alexander Zarbock

**Affiliations:** 1 Department of Anesthesiology, Intensive Care and Pain Medicine, University Hospital Muenster, Muenster, 48149 Germany; 2 Department of Pediatrics, School of Medicine in St. Louis, Washington University, St. Louis, USA; 3 Department of Pathology and Laboratory Medicine (UCSF), San Francisco, USA; 4 Department of Experimental Pathology/Center for Molecular Biology of Inflammation (ZMBE), University Muenster, Muenster, 48149 Germany; 5 Institute of Immunology, University Muenster, Muenster, 48149 Germany; 6 Institute for Clinical and Experimental Transfusion Medicine, University Hospital Tuebingen, Tuebingen, Germany

**Keywords:** STIM, ORAI, SOCE, Calcium, CD11a, CD11b, Selectin, Chemokine, Neutrophil, Inflammation

## Abstract

**Background:**

Impaired integrin activation on neutrophils is the hallmark of leukocyte adhesion deficiency syndrome in humans, characterized by reduced leukocyte recruitment. The regulation of intracellular calcium levels in neutrophils is important for cellular processes; however, the exact role of store-operated calcium entry (SOCE) and the involvement of stromal interaction molecule (STIM) calcium sensors, and ORAI1-3 calcium channels in neutrophil activation and recruitment is unknown.

**Methods:**

Using an acute kidney injury (AKI) model, intravital microscopy, and biochemical studies, we examined the molecular mechanisms of Ca^2+^-regulated neutrophil activation and recruitment.

**Results:**

We demonstrate that STIM1 and ORAI1 in neutrophils are selectively required for E-selectin- and CXCL-1-, but not P-selectin-mediated activation of the β_2_-integrin CD11a and neutrophil recruitment. Surprisingly, we did not detect an impact of STIM and ORAI isoform expression on neutrophil CD11b activation. Using a clinically relevant murine AKI model, we point out that STIM1- and ORAI1-deficiency in neutrophils prevents neutrophil recruitment into the kidney during sterile inflammation.

**Conclusion:**

Thus, we uncover stimulus specific integrin regulation in neutrophils as a critical determinant of an adequate immune response and pinpoint the clinical relevance of STIM1 and ORAI1.

**Supplementary Information:**

The online version contains supplementary material available at 10.1186/s12964-026-02666-w.

## Summary

STIM1 and ORAI1 are crucial for E-selectin- and CXCL-1-induced CD11a activation in neutrophils. Surprisingly, P-selectin-dependent signaling is unaffected. STIM1 and ORAI1 are important for neutrophil recruitment following sterile tissue injury and have severe impact on organ function.

## Background

Neutrophils are key players in pathological inflammation and many different clinical diseases [1]. As a response to tissue injury, the release of chemokines and other pro-inflammatory mediators induces inflammation characterized by the recruitment of innate effector cells into the damaged tissue [2]. Excessive neutrophil accumulation in sterile inflammation is responsible for the immunopathology observed in many diseases, including autoimmunity, trauma, and ischemic injury [3]. Therefore, understanding the mechanisms of neutrophil recruitment is of major physiological and pathophysiological significance.

Store-operated calcium entry (SOCE), a primary mechanism of calcium (Ca^2+^) influx occurring in neutrophils, is crucial for the regulation of neutrophil activity. Modulation of intracellular Ca^2+^ levels is indispensable for cellular processes, including adhesion, migration, as well as the activation of distinct adhesion molecules [4–7]. Ca^2+^ is a well-suited second messenger and is either sequestered outside the cell or within organelles including the endoplasmic reticulum (ER) [6]. Stimulation of G-protein coupled receptors (GPCRs) or P-selectin glycoprotein ligand-1 (PSGL-1) engagement on neutrophils initiates a signaling cascade [8] resulting in the production of diacylglycerol (DAG) and inositol triphosphate (IP_3_) from phosphatidylinositol 4,5-bisphosphate [9]. Ligation of IP_3_ to the IP_3_ receptor on the ER triggers Ca^2+^ release from the ER resulting in an elevated cytosolic calcium concentration [10]. Stromal interaction molecules (STIM1 and STIM2), located in the ER membrane, sense the depletion of calcium and undergo an oligomerization and conformational change to open calcium-release activated calcium (CRAC; ORAI1, ORAI2 and ORAI3) channels in the plasma membrane [11]. CRAC channel opening induces rapid calcium entry and activation of calcium-mediated intracellular signaling pathways [9]. Neutrophil recruitment, cytoskeletal rearrangement, and β_2_‐integrin activation are calcium signaling‐dependent processes [4, 5].

Integrins are heterodimeric transmembrane receptors consisting of α- and β-subunits, providing stable adhesion to other cells or to the extracellular matrix. Integrins also act as signaling platforms (outside-in signaling), triggering cell polarity, migration, proliferation, and survival [12]. As integrins reside in an inactive state in resting cells, activation is required for integrin-ligand binding (inside-out signaling) [13]. Neutrophil recruitment to sites of inflammation requires adequate activation of β_2_-integrins. The interaction of endothelial P- and E-selectin with PSGL-1 expressed on neutrophils induces the activation of CD11a into the extended conformation enabling binding to intercellular adhesion molecule 1 (ICAM-1) on inflamed endothelium [14–16]. During rolling, neutrophils are exposed to chemokines, including CXCL-1, presented on inflamed endothelium. GPCR ligation induces opening of the integrin headpiece, promoting the integrin high-affinity state and neutrophil firm adhesion [17, 18]. Following adhesion, neutrophils crawl along the endothelium, a process dependent on β_2_-integrin macrophage antigen-1 (Mac-1, CD11b) [14, 19, 20]. Integrin activation may also induce neutrophil functional responses, including migration, degranulation, and reactive oxygen species (ROS) production [21].

The importance of STIM and ORAI proteins in inflammatory responses and outside-in signaling has already been investigated [4–6, 9, 22]. However, little is known about their possible role in fine-tuning the different steps of neutrophil recruitment, in inside-out signaling, and downstream signaling mechanisms following SOCE. Using different functional *in vivo*, *ex vivo* and *in vitro* assays, we report that STIM1 and ORAI1 are required for CXCL-1 and E-selectin, but not P-selectin triggered CD11a activation during neutrophil recruitment. Therefore, we demonstrate that distinct STIM and ORAI isoforms are indispensable for an adequate and adapted neutrophil-mediated immune response to varying inflammatory stimuli.

## Methods

### Mice

STIM1^fl/fl^, STIM2^fl/fl^, STIM1/2^fl/fl^, ORAI1^fl/fl^ and ORAI2^fl/fl^ were a gift from Clifford Lowell (Department of Pathology and Laboratory Medicine, San Francisco) and have been previously described [22–25]. Single- and double mutant (STIM1/2^fl/fl^ and STIM1/ORAI1^fl/fl^) mice were bred to mice bearing transgenes with Cre recombinase under the control of *LysM-Cre* promoters [26]. Control mice were littermate (flox/flox) mice. 8–12 week-old STIM1^LysM-Cre+^, STIM2^LysM-Cre+^, STIM1/2^LysM-Cre+^, ORAI1^LysM-Cre+^, ORAI2^LysM-Cre+^, STIM1/ORAI1^LysM-Cre+^ mice, LysM^cre+^ [26], C57BL/6 (Charles River Laboratories, Wilmington, Massachusetts, USA) and Rap1a^-/-^ [27, 28] mice were used throughout this study. Animals were maintained in a special pathogen-free facility at the University of Muenster. All animal experiments were approved by the animal research committee of the Landesamt für Verbraucherschutz und Ernährung (LAVE) (G19.A182, G24-395).

### Flow chamber systems

In order to investigate the rolling velocity of murine neutrophils on E-selectin and P-selectin, we used a previously described flow chamber system [29]. Rectangular glass capillaries (20 × 200 µm) were filled either with E-selectin (2.5 µg/ml, R&D Systems), P-selectin (17 µg/ml, R&D Systems) alone or in combination with ICAM-1 (2 µg/ml and 4 µg/ml, R&D Systems) for 2 h and followed by blocking for 2 h using casein (1%, Pierce Chemicals). One side of the chamber was connected to a PE 10 tubing (BD) and inserted into a mouse carotid artery. The other side of the chamber was connected to a water-filled PE 50 (BD) tubing and used to control the wall shear stress in the capillary. Rolling velocity was analyzed using ImageJ (version v.1.54 h). Chemokine-induced adhesion *in vitro* was performed by coating glass capillaries with 50 μg/ml P-selectin (R&D Systems), 15 μg/ml ICAM-1 (R&D Systems) and 25 μg/ml CXCL-1 (Peprotech) for 2 h and then blocking for 1 h using casein (1%, Pierce Chemicals). Following an initial perfusion period of 5 min, 5–10 representative fields of view were recorded, and the number of adherent cells was determined [30].

### Biochemical experiments

Determination of STIM and ORAI isoform expression was performed by Western blot analysis. Isolated bone-marrow derived neutrophils were lysed in RIPA buffer and boiled with Laemmli sample buffer (10 min, 95 °C). Cell lysates were run in 10% SDS-PAGE and immunoblotted using antibodies against STIM1 (Cell Signaling), ORAI1 (Invitrogen), STIM2 (Cell Signaling), ORAI2 (Proteintech) and GAPDH (Cell Signaling). Determination of Rap1 activity was performed by using a Rap1 pull down assay (Thermo Fisher Scientific) according to the manufacturer’s protocol. Briefly, bone-marrow derived murine neutrophils were isolated and suspended in PBS (containing 1 mM CaCl_2_ and MgCl_2_). The cells were plated on E-selectin (3 µg/ml) or P-selectin (5 µg/ml) coated dishes or for 10 min under rotating conditions, in the presence of CXCL-1 (100 ng/ml, 37 °C, Peprotech) or left unstimulated. Neutrophils were then lysed in ice-cold EDTA-free lysis buffer for 5 min. Subsequently, cell lysates were incubated with GST-RalGDS-RBD (Thermo Fisher Scientific) for 1 h at 4 °C. Afterwards, lysates were boiled with sample buffer (5 min, 95 °C), run on 12% SDS-PAGE, and immunoblotted using antibodies against Rap1. Immunoblots were developed using an ECL system (GE Healthcare). Densitometric quantification was performed using ImageJ software (version v.1.54 h).

### Measurement of intracellular calcium levels

The measurement of intracellular calcium levels of isolated murine bone marrow neutrophils before and following CXCL-1 (100 ng/ml) stimulation was performed as described previously [22, 31]. A cell suspension (10^7^ cells/ml) was resuspended in Hanks balanced salt solution (HBSS) (containing 20 mM HEPES and 1 mM each CaCl_2_ and MgCl_2_) and loaded with 2 µM Fluo-4 AM (Invitrogen, Thermo Fisher Scientific) and 2.5 mM probenecid (Invitrogen, Thermo Fisher Scientific) in the dark at RT for 45 min. Additionally, cells were labeled with Ly6.B2-APC (Bio Rad). In some experiments, cells were incubated with 5 µM BAPTA (Invitrogen, Thermo Fisher Scientific) for 10 min at RT or with 1 µM thapsigargin (Invitrogen, Thermo Fisher Scientific) followed by the stimulation with CXCL-1. Cells were analyzed via flow cytometry (BD Accuri C6). The kinetic of the mean fluorescent intensity was calculated using FlowJo (version 10.8.3).

### Soluble ICAM-1- and fibrinogen-binding assay

The soluble ICAM-1- and fibrinogen-binding assays were performed as previously described [32]. To assess ICAM-1 binding, isolated murine neutrophils were preincubated with a blocking anti-CD11b (clone M1/70, 10 μg/ml) antibody to prevent CD11b-dependent ICAM-1 binding, or in some control experiments with a functional blocking anti-CD11a (clone TIB-217, 10 μg/ml) antibody to prevent CD11a-dependent ICAM-1 binding, Afterwards, neutrophils were stimulated with either CXCL-1 (100 ng/ml, 3 min, 37 °C) or left unstimulated in the presence of ICAM-1/Fc (20 μg/ml, R&D Systems) and APC-conjugated anti-human IgG1 (Fc-specific, Southern Biotechnology). Neutrophils were fixed on ice and stained with FITC-conjugated anti-Ly6B.2 antibody (clone 7/4, MCA771G, Bio-Rad). ICAM-1/Fc binding was measured by flow cytometry (BD FACSCantoII). The mean fluorescence intensities were evaluated using FlowJo (version 10.8.3). To investigate neutrophil binding to fibrinogen [33], isolated murine neutrophils were incubated for 10 min at 37 °C with 150 μg/ml Alexa 647-conjugated fibrinogen (Thermo Fisher Scientific) and stimulated with CXCL-1 or were left unstimulated. Cells were additionally stained with Ly6B.2 (clone 7/4, MCA771G, Bio-Rad). Fluorescence intensity was measured by flow cytometry (BD FACSCantoII). The percentage of neutrophils positive for fibrinogen binding was calculated by defining a threshold of the fluorescence intensity using FlowJo (version 10.8.3). For some experiments, cells were treated with 5 µM BAPTA (Invitrogen, Thermo Fisher Scientific) for 10 min, 1 µM thapsigargin (Invitrogen, Thermo Fisher Scientific).

### Intravital microscopy

Mice were anesthetized by intraperitoneal administration of ketamine (100 μg/g bw; Sanofi Winthrop Pharmaceuticals) and xylazine (10 μg/g bw; Tranqui Ved, Phonix Scientific), and the cremaster muscle was prepared for intravital imaging as previously described [29]. Postcapillary venules with a diameter of 20–40 µm were investigated. To determine leukocyte adhesion, 500 ng CXCL-1 (Peprotech) was injected via the carotid artery. The number of adherent cells prior to and following CXCL-1 injection was analyzed. To induce inflammation *in vivo*, mice were injected intrascrotally with TNF-α (500 ng, R&D Systems) 2 h before the preparation of the cremaster muscle. To investigate E-selectin-mediated slow rolling, adhesion, and transmigration *in vivo*, mice received TNF-α as well as PTx (pertussis toxin, 4 ug (i.v.), Sigma-Aldrich) 2 h before preparation of the murine cremaster. To block P-selectin, a monoclonal antibody (30 μg, clone RB40.34; BD Pharmingen) was injected via the carotid artery shortly before the experiment. Intravital microscopy was performed on an upright microscope (Axioskop; Zeiss) with a 40 × 0.75 NA saline immersion objective. Leukocyte rolling velocity and adhesion were determined by transillumination intravital microscopy, whereas leukocyte extravasation was investigated by reflected light oblique transillumination (RLOT) microscopy as previously described [34]. Recorded images were analyzed offline using ImageJ (version v.1.54 h) and SlideBook software. Emigrated cells were determined in an area 75 × 100 μm to each side of a vessel (representing 1.5 × 10^4^ μm^2^ tissue area). The microcirculation was recorded using a digital camera (Sensicam QE).

### Renal Ischemia–reperfusion injury (IRI)

The IRI model has been described previously [35]. In brief, mice were anesthetized by intraperitoneal administration of ketamine (100 μg/g body weight (bw); Sanofi Winthrop Pharmaceuticals) and xylazine (10 μg/g bw; Tranqui Ved) and were placed on a heating pad to maintain body temperature. In animals undergoing IRI, kidneys were surgically exposed and both renal pedicles were clamped off for 35 min with hemostatic micro clips. After clamp removal, kidneys were checked for a change in color within 3 min to ensure reperfusion. Incisions were closed in two layers. Animals were kept on a heating pad to maintain body temperature and had free access to food and water. After 24 h, the mice were euthanized and blood samples were taken. Kidneys were harvested to assess the number of recruited neutrophils. Neutrophil recruitment into the kidneys was determined by flow cytometry as previously described [35]. In brief, kidneys were mechanically minced and enzymatically digested by incubation with collagenase, hyaluronidase and DNaseI for 45 min at 37 °C. After 1 h, the homogenized kidney tissue was passed over a 70 µm cell strainer. Neutrophils were stained with fluorescently labeled antibodies against CD45 (clone 30-F11, BioLegend, 103,132), GR-1 (clone RB6-8C5, purified from hybridoma supernatant), and Ly6B.2 (clone 7/4, MCA771G, Bio-Rad). Samples were measured in a flow cytometer (BD FACSCantoII). Isotype controls were employed to account for nonspecific antibody binding. Neutrophils were identified as CD45^+^Gr-1^+^Ly6B.2^+^ cells. The total number of neutrophils in the samples was evaluated using fluorescent counting beads and were analyzed with FlowJo software (version 10.8.3). Plasma creatinine as well as urea nitrogen levels were determined by using a creatinine assay (Diazyme) and a urea nitrogen (BUN) Colorimetric Detection Kit (Invitrogen, Thermo Fisher Scientific) according to the manufacturer’s protocols. The ELISA plates were quantitatively analyzed on a Synergy 2 plate reader (BioTek).

### Statistics

Statistical analysis was performed by using GraphPad Prism (version 9). Differences between more than two groups were evaluated by one-way or two-way ANOVA followed by a Šídák's testing. Two-way repeated measured ANOVA was applied for more than two groups with two different categorical independent variables and additionally followed by a Šídák's multiple comparison test. The number of experimental repeats is specified in the corresponding figure legend. For *in vivo* experiments, the provided n is the total number of individual mice used per experiment group. All data are presented as mean ± SEM, and p < 0.05 was considered statistically significant.

## Results

### STIM1 and ORAI1 are required for E-selectin- but not P-selectin-dependent slow neutrophil rolling *in vitro*

Prior to the investigation of the role of STIM and ORAI proteins during neutrophil recruitment, we analyzed the expression levels of STIM and ORAI isoforms in isolated neutrophils of the respective conditional knockout strains (Supplemental Fig. 1). Mice deficient for the respective isoform showed a significant reduction compared to control mice (Supplemental Fig. 1A-N).

**Fig. 1:**
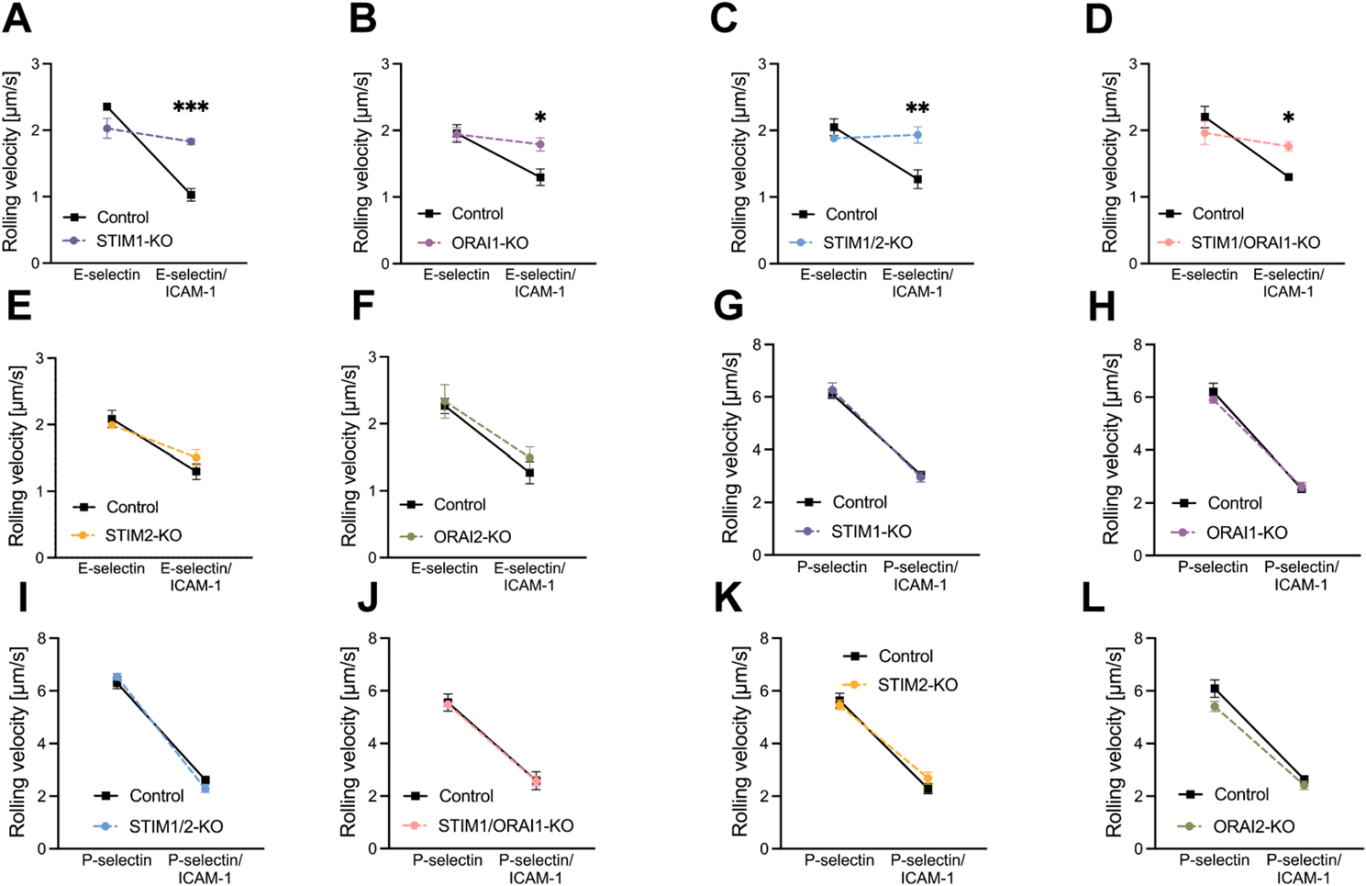
E-selectin mediated neutrophil rolling in vitro depends on STIM1 and ORAI1. The rolling velocity of control and (**A** + **G**) STIM1-KO = STIM1^LysM−Cre+^, (**B** + **H**) ORAI1-KO = ORAI1^LysM−Cre+^, (**C** + **I**) STIM1/2-KO = STIM1/2^LysM−Cre+^ and (**D** + **J**) STIM1/ORAI1-KO = STIM1/ORAI1^LysM−Cre+^, (**E** + **K**) STIM2-KO = STIM2^LysM−Cre+^, (**F** + **L**) ORAI2-KO = ORAI2^LysM−Cre+^ neutrophils on **(A**-**F)** E-selectin alone and E-selectin/ICAM-1 as well as **(G**-**L)** on P-selectin alone and P-selectin/ICAM-1 was analyzed via autoperfused flow chamber, *n* = 3–4, number of mice. Data are mean ± SEM.*p < 0.05, **p < 0.01, ***p < 0.001 by one-way ANOVA

To investigate the role of the STIM and ORAI isoforms in different steps of neutrophil recruitment, we performed *ex vivo* autoperfused flow chamber experiments [29, 31]. The rolling velocity of control neutrophils on E-selectin coated flow chambers was 2.35 ± 0.06 µm/s and decreased to 1.02 ± 0.16 µm/s on E-selectin and ICAM-1 (Fig. 1A). Neutrophils of STIM1- and ORAI1-conditional knockout mice presented similar rolling velocities on E-selectin (2.02 ± 0.25 µm/s, 1.94 ± 0.16 µm/s), but displayed a significantly faster rolling velocity on E-selectin and ICAM-1 (1.82 ± 0.07 µm/s, 1.79 ± 0.17 µm/s) compared to the corresponding control neutrophils (Fig. 1A + B). Similar results were detected in the absence of STIM1/2 and STIM1/ORAI1 neutrophils (Fig. 1C + D). Rolling velocities of STIM2- and ORAI2-deficient neutrophils on E-selectin and E-selectin/ICAM-1 were not affected (Fig. 1E + F). We also employed the autoperfused flow chamber system to explore P-selectin-mediated rolling [29]. The rolling velocity of control neutrophils on P-selectin alone was 6.10 ± 0.28 µm/s and decreased to 3.05 ± 0.06 µm/s on P-selectin and ICAM-1 (Fig. 1G). However, the specific elimination of STIM and/or ORAI molecules in neutrophils did not affect the velocity reduction on P-selectin and ICAM-1 (Fig. 1G-L), suggesting that STIM1 and ORAI1 are specifically involved in E-selectin-mediated neutrophil rolling, but not P-selectin-mediated neutrophil rolling.

### CXCL-1-induced neutrophil adhesion *in vitro* depends on STIM1 and ORAI1

**Fig. 2:**
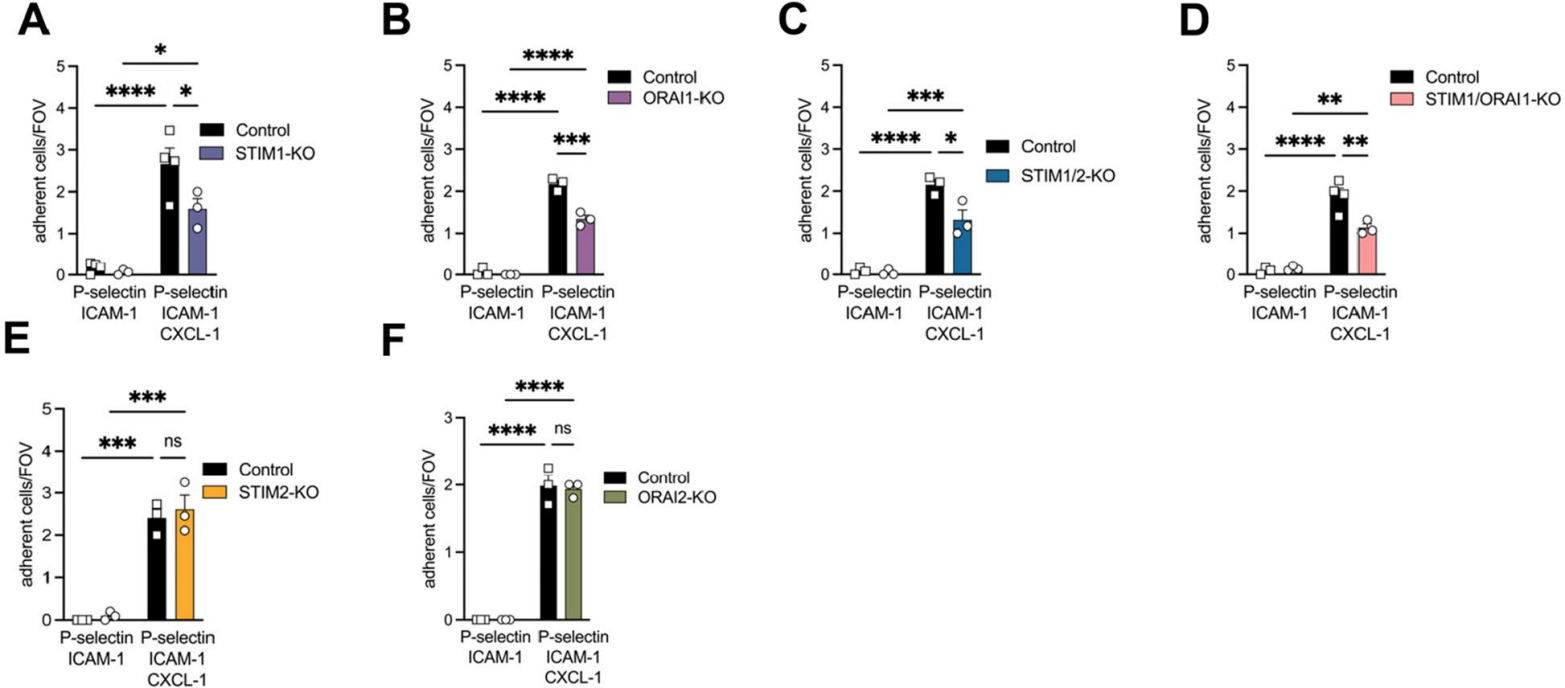
CXCL-1 induced neutrophil adhesion depends on STIM1 and ORAI1 *in vitro*. Adhesion of control and (**A**) STIM1-KO = STIM1^LysM−Cre+^, (**B**) ORAI1-KO = ORAI1^LysM−Cre+^, (**C**) STIM1/2-KO = STIM1/2^LysM−Cre+^, (**D**) STIM1/ORAI1-KO = STIM1/ORAI1^LysM−Cre+^, (**E**) STIM2-KO = STIM2^LysM−Cre+^ and (**F**) ORAI2-KO = ORAI2^LysM−Cre+^ neutrophils on *P*-selectin/ICAM-1 or P-selectin/ICAM-1 and CXCL-1 was determined via autoperfused flow chamber, *n* = 3–4, number of mice. Data are mean ± SEM. *p < 0.05, **p < 0.01, ***p < 0.001, ns = non significant by one-way ANOVA

In order to investigate chemokine-mediated neutrophil arrest, we quantified neutrophil adhesion to flow chambers either coated with P-selectin- and ICAM-1 or P-selectin, ICAM-1 and CXCL-1 [31]. Here, we demonstrate that neutrophils lacking STIM1, ORAI1, STIM1/2, and STIM1/ORAI1 adhered significantly less to P-selectin, ICAM-1 and CXCL-1 coated flow chambers compared to control neutrophils (Fig. 2A-D). Adhesion of STIM2- or ORAI2-deficient neutrophils was not affected (Fig. 2E + F). These data suggest, that STIM1 and ORAI1 are involved in CXCL-1-induced neutrophil arrest.

### STIM1 and ORAI1 are required for E-selectin-induced signaling

**Fig. 3:**
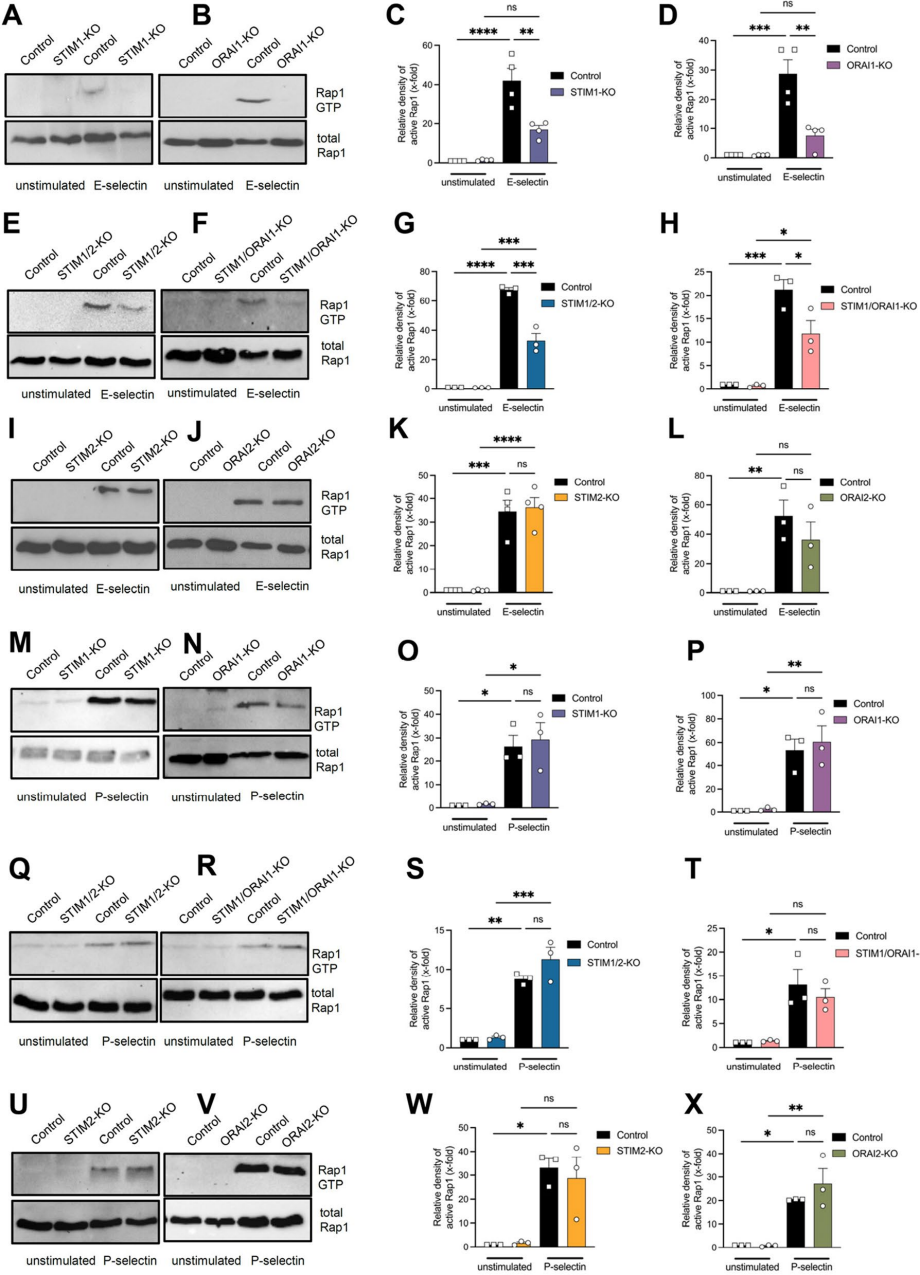
STIM1 and ORAI1 are crucial for E-selectin- mediated Rap1 activation. Determination of Rap1 activation via pull-down assay from control and (**A**, **C**, **M**, **O**) STIM1-KO = STIM1^LysM−Cre+^, (**B**, **D**, **N**, **P**) ORAI1-KO = ORAI1^LysM−Cre+^, (**E**, **G**, **Q**, **S**) STIM1/2-KO = STIM1/2^LysM−Cre+^, (**F**, **H**, **R**, **T**) STIM1/ORAI1-KO = STIM1/ORAI1^LysM−Cre+^, **(I**, **K**, **U**, **W**) STIM2-KO = STIM2^LysM−Cre+^ and (**J**, **L**, **V**, **X**) ORAI2-KO = ORAI2^LysM−Cre+^ neutrophils either unstimulated or stimulated with (**A**-**L**) E-selectin or (**M**-**X**) P-selectin. (**A**–**X**) Lysates were immunoblotted with anti-Rap1, *n* = 3–4, experimental repeat. Representative western blot images are cropped. Data are mean ± SEM. *p < 0.05, **p < 0.01, ****p < 0.0001, ns = non significant by one-way ANOVA

As we demonstrated that STIM1 and ORAI1 are required for E-selectin dependent slow neutrophil rolling (Fig. 1), we further explored the role of STIM and ORAI isoforms for the E-selectin induced signaling resulting in CD11a activation. Stimulation of neutrophils with selectins induces downstream signaling via the GTPase Rap1 resulting in activation of CD11a [15, 19, 36]. In isolated control neutrophils, activation of Rap1 was increased following E-selectin stimulation, while the loss of STIM1 and ORAI1 partially abolished the activation of Rap1 (Fig. 3A-D). In STIM1/2- and STIM1ORAI1-deficient neutrophils, E-selectin-induced Rap1 activity was significantly reduced (Fig. 3E-H). E-selectin-mediated Rap1 activation in STIM2- and ORAI2-deficient neutrophils was not affected (Fig. 3I-L). Stimulating neutrophils with P-selectin increased Rap1 activity in control neutrophils. Rap1 activation in P-selectin-stimulated neutrophils was not affected in the absence of any STIM or ORAI isoform (Fig. 3M-X). These data demonstrate that E-selectin-, but not P-selectin-mediated, intracellular signaling in neutrophils is altered in STIM1- and ORAI1-deficient neutrophils.

### STIM1 and ORAI1 regulate CXCL-1-induced intracellular signaling

**Fig. 4:**
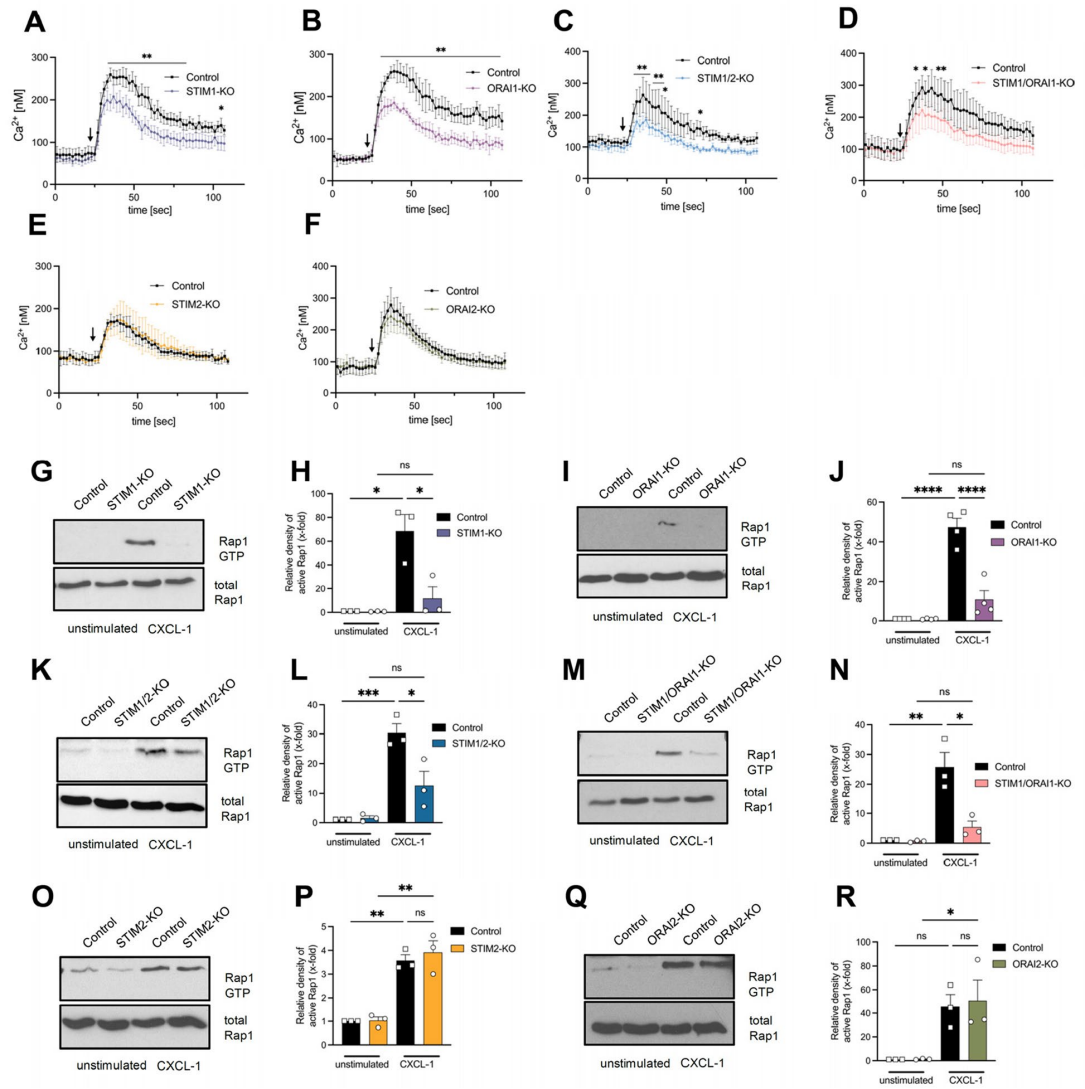
STIM1 and ORAI1 are crucial regulators of CXCL-1-mediated intracellular signaling pathways in neutrophils. Intracellular calcium levels were measured in Fluo-4 labeled control and (**A**) STIM1-KO = STIM1^LysM−Cre+^, (**B**) ORAI1-KO = ORAI1^LysM−Cre+^, (**C**) STIM1/2-KO = STIM1/2^LysM−Cre+^, (**D**) STIM1/ORAI1-KO = STIM1/ORAI1^LysM−Cre+^, (**E**) STIM2-KO = STIM2^LysM−Cre+^ and (**F**) ORAI2-KO = ORAI2^LysM−Cre+^ isolated neutrophils before and following CXCL-1 stimulation, n = 3–6, experimental repeat. Determination of Rap1 activation via pull-down assay from control and (**G**-**H**) STIM1-KO = STIM1^LysM−Cre+^, (**I**-**J**) ORAI1-KO = ORAI1^LysM−Cre+^, (**K**-**L**) STIM1/2-KO = STIM1/2^LysM−Cre+^, (**M**–**N**) STIM1/ORAI1-KO = STIM1/ORAI1^LysM−Cre+^, (**O**-**P**) STIM2-KO = STIM2^LysM−Cre+^ and (**Q**-**R**) ORAI2-KO = ORAI2^LysM−Cre+^ neutrophils either unstimulated or stimulated with CXCL-1. (**G**-**R**) Lysates were immunoblotted with anti-Rap1, *n* = 3–4, experimental repeat. Representative Western blot images are cropped. Data are mean ± SEM. *p < 0.05, **p < 0.01, ****p < 0.0001, ns = non significant by one-way ANOVA

To assess the role of STIM and ORAI isoforms in CXCR2-induced neutrophil SOCE, we analyzed calcium influx downstream of neutrophil CXCL-1 ligation [9]. CXCL-1 stimulation of control neutrophils resulted in an increased intracellular calcium concentration (Fig. 4A-F). The loss of STIM1 and ORAI1 impaired intracellular calcium levels compared to control neutrophils (Fig. 4A + B). Corresponding results were obtained in STIM1/2- and STIM1/ORAI1-deficient neutrophils, while the absence of STIM2 and ORAI2 did not affect calcium influx (Fig. 4C-F). These data suggest that ORAI1 and STIM1 are essential for CXCR2-mediated neutrophil SOCE. To elucidate the outstanding function of STIM1 and ORAI1 in CXCL-1-induced downstream signaling, we examined the activation of the GTPase Rap1. Following CXCL-1 stimulation, the activation of Rap1 was increased in isolated control neutrophils (Fig. 4G-R). The loss of STIM1 and ORAI1 significantly abrogated CXCL-1-induced Rap1 activation (Fig. 4G-J). Corresponding results were observed in the absence of STIM1/2 and STIM1/ORAI1 in neutrophils (Fig. 4K-N). Neutrophil STIM2 and ORAI2 are not involved in Rap1 activation after stimulation with CXCL-1 (Fig. 4O-R). These data suggest that CXCL-1-induced signaling, resulting in CD11a activation, is STIM1 and ORAI1 dependent.

### Calcium influx via STIM1 and ORAI1 is required for CXCL-1-induced CD11a, but not CD11b activation in neutrophils

**Fig. 5:**
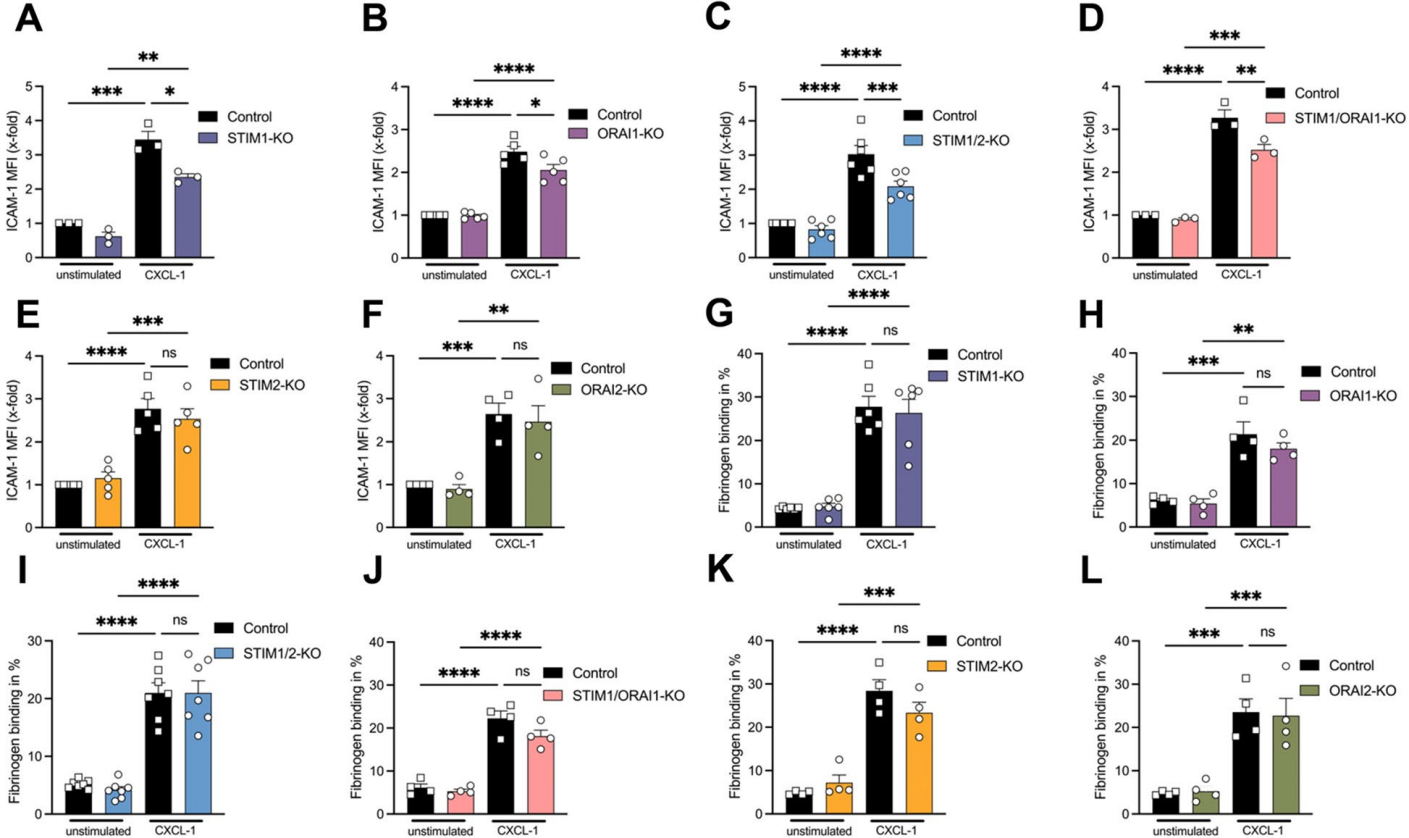
CXCL-1 induced CD11a activation depends on STIM1 and ORAI1. Binding of fluorescently coupled β_2_-integrin ligands (**A**-**F**) ICAM-1 and (**G**-**L**) fibrinogen to either unstimulated or CXCL-1-stimulated isolated control and (**A**, **G**) STIM1-KO = STIM1^LysM−Cre+^, (**B**,** H**) ORAI1-KO = ORAI1^LysM−Cre+^, (**C**, **I**) STIM1/2-KO = STIM1/2^LysM−Cre+^, (**D**, **J**) STIM1/ORAI1-KO = STIM1/ORAI1^LysM−Cre+^, (**E**, **K**) STIM2-KO = STIM2^LysM−Cre+^ and (**F**, **L**) ORAI2-KO = ORAI2^LysM−Cre+^ neutrophils was assessed by flow cytometry, *n* = 3–7, experimental repeat. Data are mean ± SEM. *p < 0.05, **p < 0.01, ***p < 0.001, ****p < 0.0001, ns = non significant by one-way ANOVA

To further investigate the function of the calcium-regulating proteins during β_2_-integrin activation, ligand binding assays were performed. In order to specifically investigate CD11a activation, we quantified ICAM-1 binding of isolated neutrophils in the presence of either a blocking CD11b or CD11a antibody (Supplemental Fig. 2A) [32]. After CXCL-1 stimulation, neutrophil ICAM-1 binding was significantly decreased upon exposure to a blocking CD11a antibody in comparison to control. However, a blocking CD11b antibody did not affect neutrophil ICAM-1 binding in comparison to control (Supplemental Fig. 2A). In the presence of a blocking CD11b antibody, deficiency of STIM1 and/or ORAI1 in neutrophils revealed a reduced ICAM-1 binding after stimulation with CXCL-1 (Fig. 5A-D). However, STIM2- or ORAI2-deficiency in neutrophils did not reduce CXCL-1-dependent ICAM-1 binding compared to control neutrophils (Fig. 5E + F). Similarly, the absence of Rap1a in neutrophils resulted in a reduced ICAM-1 binding after CXCL-1 stimulation (Supplemental Fig. 2B). These data indicate that STIM1 and ORAI1 as well as Rap1 are involved in CXCL-1-induced CD11a activation.

Furthermore, to explore CD11b activation, we analyzed fibrinogen binding via flow cytometry [13, 33, 37]. Fibrinogen binding was significantly increased following CXCL-1 stimulation of control and STIM-, ORAI-, and Rap1-deficient neutrophils (Fig. 5G-L, Supplemental Fig. 1C). These findings suggest that STIM and ORAI are not required for CXCL-1-induced fibrinogen binding to neutrophils.

To directly assess the impact of varying intracellular calcium levels in neutrophils on integrin activation, we modulated intracellular Ca^2+^ levels by using either the calcium chelator BAPTA, which depletes the intracellular free calcium ions [38], or thapsigargin, which raises intracellular calcium concentration via inhibition of the sarcoplasmic-ER calcium ATPase [39, 40]. CXCL-1-induced SOCE was significantly reduced in BAPTA pretreated wildtype neutrophils but significantly increased after thapsigargin treatment (Supplemental Fig. 2D + E). ICAM-1 binding of BAPTA pretreated wildtype neutrophils following stimulation with CXCL-1 was decreased (Supplemental Fig. 2F). In contrast, thapsigargin pretreatment significantly increased ICAM-1 binding (Supplemental Fig. 2G). In line with our previous results, CXCL-1 induced fibrinogen binding of wildtype neutrophils was not affected by BAPTA or thapsigargin pretreatment (Supplemental Fig. 2H + I). Together, these data demonstrate that calcium is a key regulator in CXCL-1-induced CD11a, but not CD11b activation in neutrophils.

### STIM1 and ORAI1 are required for E-selectin-dependent rolling and neutrophil recruitment *in vivo*

To confirm the obtained data *in vivo*, we performed intravital microscopy of the murine cremaster muscle. Two hours after TNF-α injection, leukocyte rolling along the endothelial surface is mediated by P- and E-selectin expressed on inflamed endothelial cells [17, 29]. STIM1 and ORAI1 deficient mice exhibit an increased neutrophil rolling velocity compared to control mice (Supplemental Fig. 3A + B). Neutrophil rolling velocity in the absence of STIM1/2, STIM1/ORAI1, STIM2 or ORAI2 did not differ from control neutrophils (Supplemental Fig. 3C-F). Both, selectin and GPCR signaling mediate leukocyte intravascular adhesion in venules and emigration into the surrounding tissue of the cremaster muscle after TNF-α treatment [17, 29]. The number of adherent cells and emigrated leukocytes into the surrounding tissues was significantly reduced in STIM1- and ORAI1-conditional knockout mice compared to controls (Supplemental Fig. 3G-J). Consequently, the absence of STIM1/2 or STIM1/ORAI1 also resulted in fewer adherent and emigrated leukocytes (Supplemental Fig. 3K-N). The number of adherent and transmigrated neutrophils deficient for STIM2 and ORAI2 was not affected compared to WT neutrophils (Supplemental Fig. 3O-R).

**Fig. 6:**
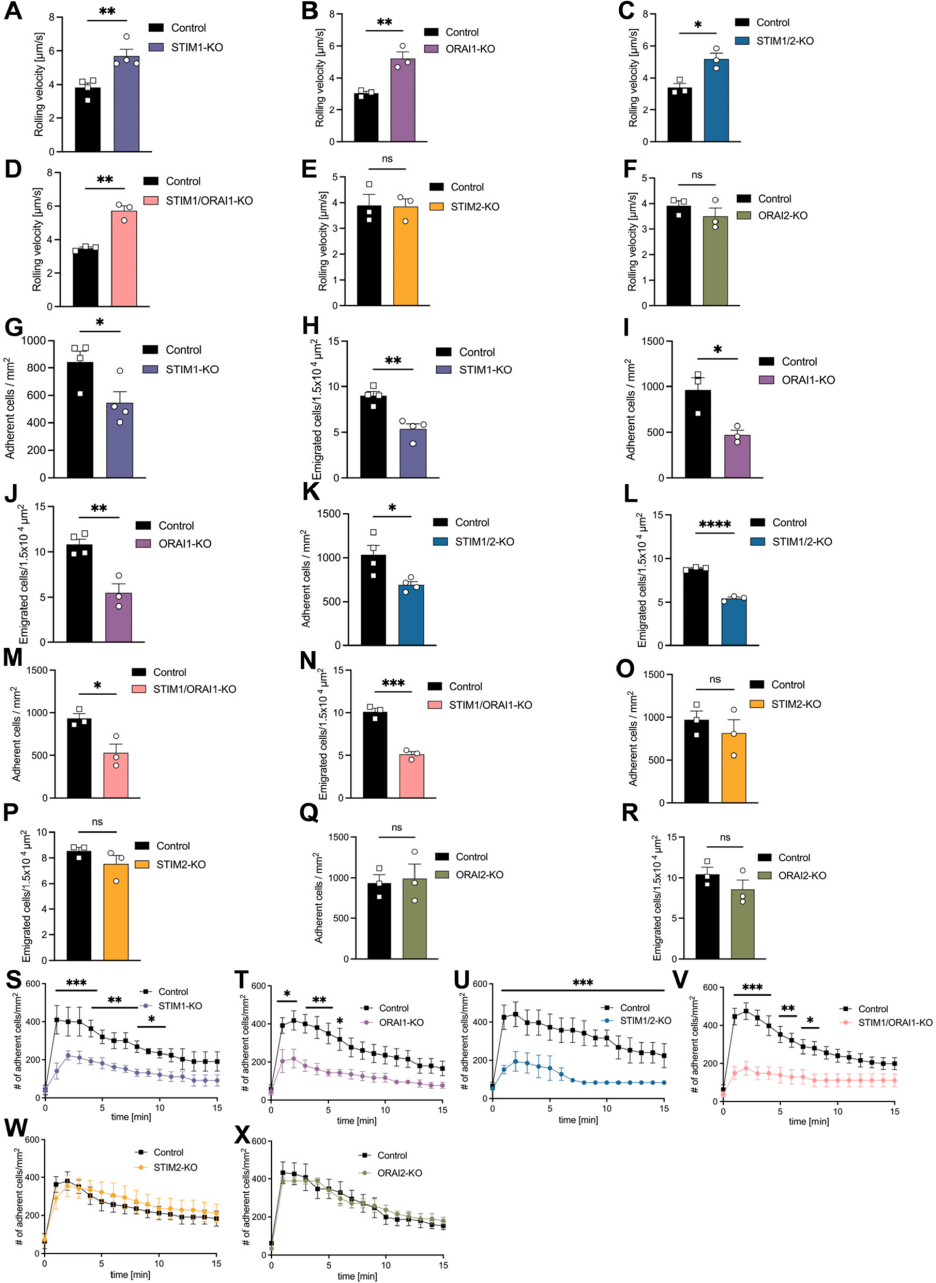
STIM1 and ORAI1 are crucial for E-selectin- and CXCL-1-induced neutrophil recruitment *in vivo.* Control and (**A**, **G**, **H**) STIM1-KO = STIM1^LysM−Cre+^, (**B**, **I**, **J**) ORAI1-KO = ORAI1^LysM−Cre+^, (**C**,** K**,** L**) STIM1/2-KO = STIM1/2^LysM−Cre+^, (**D**,** M**,** N**) STIM1/ORAI1-KO = STIM1/ORAI1^LysM−Cre+^, (**E**, **O**, **P**) STIM2-KO = STIM2^LysM−Cre+^ and (**F**, **Q**, **R**) ORAI2-KO = ORAI2^LysM−Cre+^ mice were treated with PTx and a blocking P-selectin antibody RB40.34 Two hours after intrascrotal TNF-α injection, neutrophil recruitment was analyzed. (**A**-**F**) Rolling velocity and (**G**,** I**, **K**, **M**,** O**,** Q**) the number of adherent cells was analyzed in postcapillary cremaster venules. (**H**,** J**,** L**,** N**,** P**,** R**) Neutrophil transmigration was visualized using reflected light oblique transillumination microscopy (RLOT), *n* = 3–4, number of mice. In control and (**S**) STIM1-KO = STIM1^LysM−Cre+^, (**T**) ORAI1-KO = ORAI1^LysM−Cre+^, (**U**) STIM1/2-KO = STIM1/2^LysM−Cre+^, (**V**) STIM1/ORAI1-KO = STIM1/ORAI1^LysM−Cre+^, (**W**) STIM2-KO = STIM2^LysM−Cre+^ and (**X**) ORAI2-KO = ORAI2^LysM−Cre+^ mice, the number of adherent cells in postcapillary cremaster muscle venules were determined prior and following CXCL-1 and injection via the carotid artery, *n* = 3–4. Data are mean ± SEM.*p < 0.05, **p < 0.01, ***p < 0.001, ns = non significant by (**A**-**R**) Student’s t-test and (**S**-**X**) by one-way ANOVA

To specifically explore E-selectin dependent rolling velocity, mice were pretreated with pertussis toxin (PT_x_) in order to block Gα_i_-signaling as well as a monoclonal blocking antibody against P-selectin [17]. The loss of STIM1 or ORAI1 in neutrophils significantly increased E-selectin-mediated *in vivo* rolling in comparison to control neutrophils (Fig. 6A + B). Similar results were observed for STIM1/2- and STIM1/ORAI1-deficient neutrophils compared to the control (Fig. 6C + D). E-selectin-mediated *in vivo* rolling of STIM2- or ORAI2-deficient neutrophils was similar to control neutrophils (Fig. 6E + F). In the absence of GPCR signaling, E-selectin mediates leukocyte intravascular adhesion and emigration into the surrounding tissue of the cremaster muscle after TNF-α treatment [17, 29]. The number of adherent and emigrated neutrophils into the surrounding tissues was significantly reduced in STIM1- or ORAI1-conditional knockout mice compared to control mice (Fig. 6G-J). In mice lacking STIM1/2 and STIM1/ORAI1, the number of adherent and emigrated neutrophils was reduced compared to the controls (Fig. 6K-N). STIM2- and ORAI2-deficiency did not affect the number of adherent and transmigrated neutrophils in this experimental setup (Fig. 6O-R). These data indicate that STIM1 and ORAI1 play a crucial role in regulating E-selectin-mediated neutrophil recruitment.

To exclude an impact of the genetically modification of the used floxed control mice on our obtained results, we performed two key control experiments using Orai1LysM^cre−^, Orai1LysM^Cre+^ and LysM^cre+^ mice. During intravital microscopy of the inflamed cremaster muscle following PTx injection as well as P-selectin blocking, we did not observe any differences in neutrophil rolling velocity, neutrophil adhesion and transmigration between LysM^+^ and Orai1LysM^cre−^ mice (Supplemental Fig. 3S-U). Furthermore, neutrophils of Orai1LysM^cre+^ mice rolled significantly faster and adhered and transmigrated significantly less than neutrophils of both control mouse strains, LysM^cre+^ and Orai1LysM^cre−^. To confirm these results also *in vitro*, we quantified CXCL-1 induced neutrophil ICAM-binding via flow cytometry. ICAM-1 binding did not differ between isolated neutrophils of floxed control mice lacking Cre recombinase expression and LysM^cre+^ mice expressing Cre recombinase in neutrophils (Supplemental Fig. 3V). CXCL-1 stimulated Orai1LysM^cre+^ neutrophils bound significantly less ICAM-1 compared to both control groups (Supplemental Fig. 3V).

In order to elucidate whether STIM and ORAI molecules are involved in GPCR-induced arrest *in vivo*, we performed intravital microscopy of the murine cremaster muscle before and after CXCL-1 injection via the carotid artery [31, 41]. In control mice, the number of adherent cells immediately increases following CXCL-1 injection (Fig. 6S-X). The number of adherent neutrophils in postcapillary cremaster muscle venules after CXCL-1 injection was significantly decreased in the absence of STIM1 or ORAI1 compared to control mice (Fig. 6S + T). Consequently, the number of adherent neutrophils in STIM1/2- and STIM1/ORAI1-conditional knockout mice was also reduced after CXCL-1 injection (Fig. 6U + V). CXCL-1-mediated adhesion in STIM2- and ORAI2-conditional knockout mice was not affected compared to control mice (Fig. 6W + X). These data indicate, that STIM1 and ORAI1 are involved in CXCL-1-induced neutrophil arrest mediated by CD11a.

### STIM1 and ORAI1 deficiency attenuates neutrophil recruitment and improves kidney function after IRI-induced AKI

**Fig. 7:**
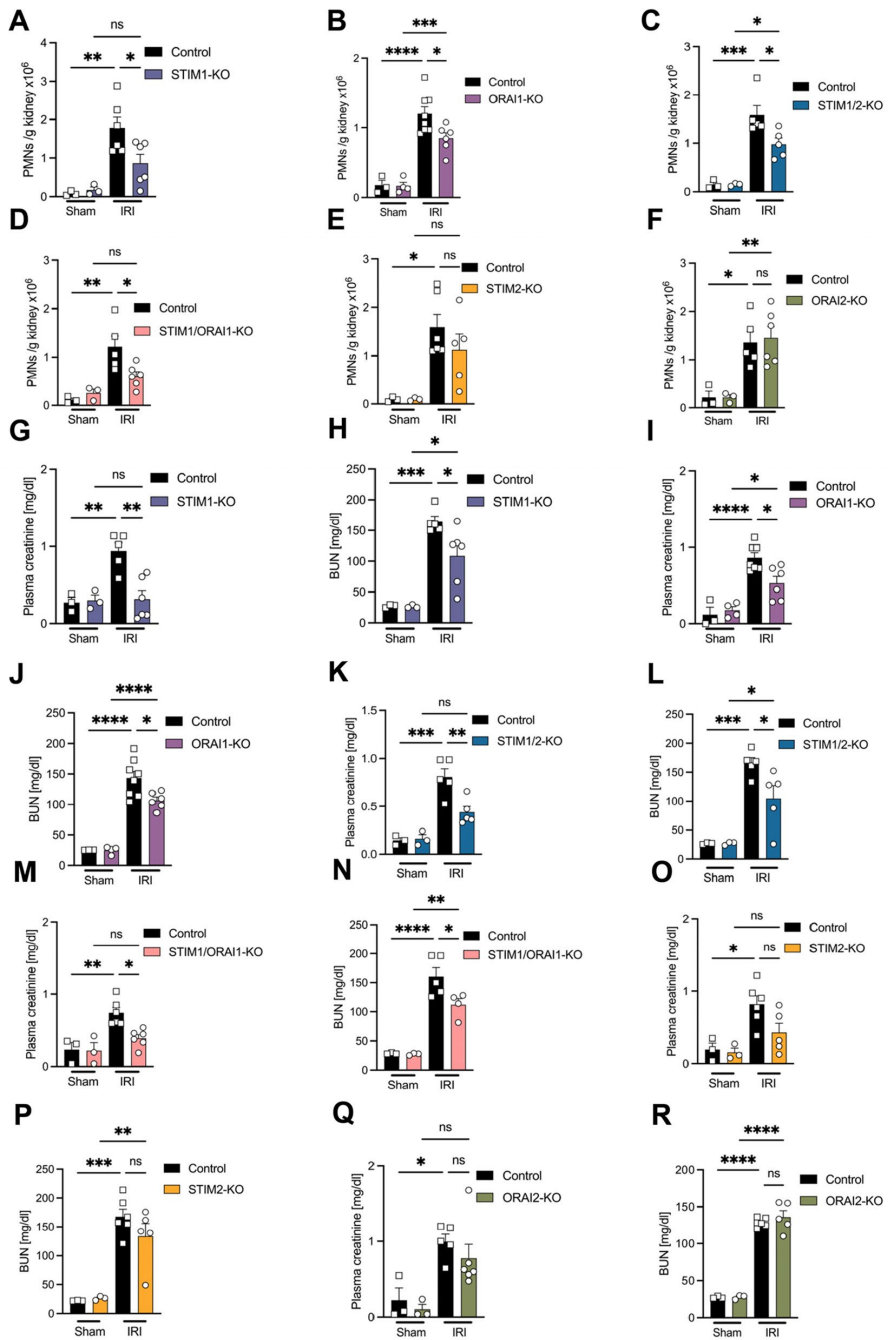
STIM1 and ORAI1 deficiency prevents neutrophil recruitment and kidney damage after IRI-induced AKI. AKI in control and (**A**, **G**, **H**) STIM1-KO = STIM1^LysM−Cre+^, (**B**, **I**, **J**) ORAI1-KO = ORAI1^LysM−Cre+^, (**C**, **K**, **L**) STIM1/2-KO = STIM1/2^LysM−Cre+^, (**D**, **M**, **N**) STIM1/ORAI1-KO = STIM1/ORAI1^LysM−Cre+^, (**E**,** O**,** P**) STIM2-KO = STIM2^LysM−Cre+^ and (**F**,** Q**,** R**) ORAI2-KO = ORAI2^LysM−Cre+^ mice was induced by IRI. Sham surgery was performed as control. (**A**-**F**) The number of recruited neutrophils (PMNs) into the kidney was determined by flow cytometry. (**G**, **I**, **K**, **M**,** O**,** Q**) plasma creatinine and (**H**, **J**, **L**, **N**,** P**,** R**) blood urea nitrogen (BUN), specific markers for kidney dysfunction, were analyzed via photometry 24 h post AKI induced by IRI. *n* = 3–6, number of mice. Data are mean ± SEM. *p < 0.05, **p < 0.01, ****p < 0.0001 ns = non significant by one-way ANOVA

To specifically investigate the role of STIM and ORAI isoforms in neutrophils during sterile inflammation, we used a clinically relevant murine model of renal IRI [31, 35]. Mice were subjected to sham or renal IRI surgery and were analyzed 24 h after reperfusion. IRI-induced neutrophil recruitment into the kidney was significantly reduced in STIM1- and ORAI1-conditional knockout mice compared to control mice (Fig. 7A + B). In STIM1/2- and STIM1/ORAI1- conditional knockout mice, neutrophil recruitment was significantly decreased in comparison to respective control mice (Fig. 7C + D). In line with the previous results, neutrophil recruitment into the kidneys was not affected in STIM2- and ORAI2- conditional knockout mice (Fig. 7E + F). After renal IRI, levels of plasma creatinine and blood urea nitrogen (BUN), markers of kidney function, were significantly reduced in the absence of neutrophil STIM1 and ORAI1 (Fig. 7G-N). However, plasma creatinine and BUN levels after renal IRI were not affected in STIM2- and ORAI2- conditional knockout mice compared to control mice (Fig. 7O-R). These data indicate that STIM1 and ORAI1 in neutrophils are crucial for the inflammatory reaction following sterile tissue injury and additionally have severe impact on the maintenance of organ function.

## Discussion

Neutrophil recruitment into damaged tissues is a complex and tightly regulated process [14, 42]. Various cellular pathways are involved in the regulation of neutrophil recruitment and inflammation. While the second messenger Ca^2+^, and in neutrophils especially the regulation of intracellular calcium concentrations via SOCE, are important for many aspects of neutrophil function, the specific role and relevance during neutrophil recruitment remain still unknown [25, 43, 44]. In this study, we demonstrate for the first time a stimulus-specific involvement of STIM1 and ORAI1 in activation CD11a-, but not CD11b-dependent inflammatory response of neutrophils as well as the involvement of STIM1- and ORAI1-regulated SOCE in neutrophils as a mediator in sterile inflammation and tissue damage.

We determined an impact of STIM1 and ORAI1 expression in neutrophils on CXCL-1- and E-selectin-induced CD11a activation via *ex vivo* flow chamber assays. A decreased neutrophil adherence in the absence of STIM1 and/or ORAI1 in neutrophils during CXCL-1-induced arrest, and an impaired slow neutrophil rolling of STIM1- as well as ORAI1-deficient neutrophils on E-selectin and the CD11a ligand ICAM-1 was observed. Surprisingly, neutrophil slow rolling on P-selectin and ICAM-1 did not depend on STIM1 and ORAI1, indicating for the first time a divergent role of STIM- and ORAI-isoforms in selectin mediated signaling pathways. Supporting our observations, Dixit and colleagues show that ORAI1 mediated calcium flux is involved in E-selectin mediated neutrophil rolling and accompanied with an increased kindlin-3 and talin-1 density at focal sites of adhesion [5]. This suggests a crucial role of ORAI1-induced SOCE mediating actin cytoskeleton rearrangements resulting in a recruitment of kindlin-3 and talin-1 and the initiation of CD11a/ICAM-1 bonds [5]. Consistently, Schaff et al. demonstrate that downregulation of kindlin-3 or ORAI1 impairs CD11a/ICAM-1 bonds [45]. Several studies demonstrate that calcium and DAG bind to and activate CalDAG-GEFI, followed by a p38 MAPK-mediated Rap1a activation [15, 36]. Here, we demonstrate that STIM1 and ORAI1 are necessary for CXCL-1 and E-selectin dependent Rap1 activation and regulate CD11a inside-out activation. Interestingly, our data demonstrate that specifically E-selectin-, but not P-selectin-induced signaling is mediated via STIM1 and ORAI1. CD11a activation may be also triggered by P-selectin-PSGL-1 ligation [46]. Kuwano et al. report that signaling downstream of PSGL-1 binding to either E-selectin or P-selectin has many identical signaling molecules including Fgr, Syk and PLC [47]. In contrast to our results, Yago and colleagues state that that P-selectin-induced β_2_‐integrin activation *in vitro* does not depend on Rap1a [48]. This may be due to significant differences in the used experimental methods concerning neutrophil isolation and stimulation, since neutrophil behavior and function is highly dependent on these factors.

The regulation of GPCR-induced changes in intracellular calcium levels via SOCE is described to be essentially involved in the activation of β_2_-integrins [4–6]. Current studies demonstrate that GPCR-induced calcium flux is impaired in STIM- and ORAI-deficient neutrophils [22, 25]. Our findings indicate that STIM1 and ORAI1 are involved in CXCL-1-induced SOCE. Furthermore, the results of CXCL-1 mediated neutrophil ICAM-1 binding confirm an involvement of STIM1 and ORAI1 in CXCL-1-induced CD11a activation. Our results indicate that calcium influx in general, and more specific all STIM- and ORAI-isoforms, are not required for CXCL-1-induced CD11b activation on neutrophils. It is known that CXCL-1 mediated neutrophil fibrinogen binding is not exclusively dependent on CD11b activation, since activated CD11c may also be involved in neutrophil fibrinogen-binding. Our results indicate that CXCL-1 mediated neutrophil fibrinogen-binding via activated integrins is not affected by STIM and ORAI expression. Furthermore, CD11c is mainly regulating neutrophil effector functions and neutrophil maturation than neutrophil recruitment [49, 50].

The differences within the signaling pathways resulting in β_2_-integrin activation on neutrophils observed in our study, might be caused by differential spatial distribution and cellular clustering of CXCR2, ORAI1, and CD11a. Studies by Dixit et al. point out that ORAI1 activation and ORAI1-dependent Ca^2+^ influx result in an increased spatial Ca^2+^ concentration as well as talin-1 recruitment to CD11a and ORAI1-CD11a clustering [5]. Other studies demonstrate that activation of ORAI calcium channels may be induced by two different pathways, SOCE or store-independent calcium entry (SICE), indicating a variety of possible activation mechanisms of ORAI isoforms [51, 52].

*In vivo* data from the murine cremaster model demonstrate fewer adherent and emigrated neutrophils in STIM1- and ORAI1-conditional knockout mice compared to WT mice. Decreased CXCL-1-induced arrest confirms an essential function of STIM1 and ORAI1 in CD11a activation. Previous studies support that ORAI1 is involved in neutrophil recruitment [5, 8, 53]. Double knockout mice lacking STIM1/2 and STIM1/ORAI1 display less recruited neutrophils and faster rolling velocity, suggesting that STIM1 and ORAI1 are the predominant cause for the existing phenotype. Contrastively, one study demonstrates an increased number of recruited neutrophils in STIM1/2-conditional knockout mice after TNF-α stimulation [54]. Their results are based on a different experimental setup without further supporting data.

In a clinically relevant AKI model, neutrophil recruitment into inflamed tissue is significantly decreased in STIM1- and ORAI1-conditional knockout mice. Several studies support our findings and demonstrate that ORAI1 chimeric mice exhibit impaired neutrophil recruitment into LPS-induced peritonitis [53, 55]. Moreover, a recent study demonstrates that neutrophil-specific ORAI1 inhibition reduced pancreatitis-associated acute lung injury [56]. Additional previous literature describes that STIM1 is involved in hepatic ischemia–reperfusion injury [43, 57]. Specifically, the authors show that STIM1 is involved in NADPH oxidase activation, ROS production, and phagocytosis leading to tissue injury. Our findings, together with the recent studies, demonstrate the important role of STIM1 and ORAI1 in the pathophysiology of AKI and as a potential target in prevention of AKI as well as other inflammatory diseases. Together with the *in vitro* data, we clearly demonstrate a specific role of the calcium regulators STIM1 and ORAI1 in E-selectin- as well as CXCL-1 dependent activation of the β_2_-integrin CD11a which is necessary for proper neutrophil recruitment in sterile inflammation.

We further demonstrate that STIM2 and ORAI2 are not required for neutrophil recruitment. In line, different *in vivo* and *in vitro* studies reported that neutrophil effector functions like ROS and phagocytosis are independent of STIM2 and ORAI2 [22, 38]. In agreement with our data, the study of Clemens et al. demonstrate a modestly impaired SOCE in STIM2-deficient neutrophils but also that STIM2 is not required for proper neutrophil recruitment. However, they were able to identify a completely new role of STIM2 in regulating neutrophil cytokine production [22]. Correspondingly, our data show an unaffected SOCE in the absence of STIM2 after CXCL-1 stimulation, which is supported by the knowledge that STIM2 exhibits a lower affinity for calcium and is mediating the basal intracellular calcium levels [58]. Interestingly, a recent study reports that ORAI2-deficient neurons have a diminished calcium signal-induced store depletion and are protected from calcium overload during ischemic strokes, indicating that ORAI2 mediates calcium flux in neurons [59]. Furthermore, Nakajima and colleagues identified a role of ORAI2 during inflammatory processes in the central nervous system (CNS) via astrocytes. Focusing on the murine cremaster and the kidney, we detect no evidence that ORAI2 is involved in neutrophil recruitment during sterile inflammation.

## Conclusion

Taken together, our data clearly show that calcium is selectively indispensable for E-selectin and CXCL-1-mediated CD11a activation. β_2_‐integrins are involved in many different steps during neutrophil recruitment. Therefore, the separate signaling pathways in activating CD11a and CD11b might have regulatory purposes. Moreover, we evidently present that STIM1 and ORAI1 are not crucial for P-selectin induced signaling in neutrophils, which gives us a vision of two independently regulated pathways leading to β_2_‐integrin activation, either via E-selectin or P-selectin. Further insights into how these processes are mediated may offer new approaches to specifically modulate different neutrophil functions. These findings extend the crucial role of SOCE regulation as a central mechanism of neutrophil recruitment and may serve as the basis for the development of specific therapeutic drugs to control or prevent immune diseases.

## Supplementary Information

Supplementary Material 1: Supplemental Fig. 1 Expression of Ca^2+^ isoforms in neutrophils. Determination of Ca^2+^ isoforms via immunoblotting from control and (A-B) STIM1-KO = STIM1^LysM-Cre+^, (C-D) ORAI1-KO =ORAI1^LysM-Cre+^, (E-G) STIM1/2-KO = STIM1/2^LysM-Cre+^, (H-J) STIM1/ORAI1-KO = STIM1/ORAI1^LysM-Cre+^, (K-L) STIM2-KO = STIM2^LysM-Cre+^ and (M-N) ORAI2-KO = ORAI2^LysM-Cre+^ neutrophils. Lysates were immunoblotted with (A, E, H) anti-STIM1, (C, H) anti-ORAI1, (E, K) anti-STIM2, (M) anti-ORAI2 and (A, C, E, H, K, M) anti-GAPDH,* n*=3, experimental repeat. Representative Western blot images are cropped. Data are mean ± SEM. *p<0.05, **p<0.01, ****p<0.0001 by one-way ANOVA. Supplemental Fig. 2. Calcium is required CXCL-1-induced CD11a activation. (A, B, C, F, G, H) Binding of fluorescently coupled β_2_-integrin ligands in unstimulated or CXCL-1 stimulated neutrophils. ICAM-1 binding to control neutrophils in presence of IgG or blocking anti-CD11a or anti-CD11b antibodies was assessed by flow cytometry, *n*=3, experimental repeat. In (B, C) control and Rap1a-KO = Rap1a^-/-^ neutrophils binding of fluorescently coupled β_2_-integrin ligands (B) ICAM-1 and (C) fibrinogen was assessed by flow cytometry, *n*=3-4, experimental repeat. Intracellular calcium levels were analyzed in Fluo-4 labeled control neutrophils, incubated with either DMSO, (D) BAPTA or (E) thapsigargin before and after CXCL-1 stimulation. Binding of fluorescent coupled β_2_-integrin ligands (F, G) ICAM-1 and (H, I) fibrinogen to control neutrophils, incubated with either DMSO (control), (F, H) BAPTA or (G, I) thapsigargin was assessed by flow cytometry, *n*=3-5, experimental repeat. Data are mean ± SEM. *p<0.05, **p<0.01,***p<0.001, ****p<0.0001, ns=non significant by one-way ANOVA. Supplemental Fig. 3. Neutrophil recruitment depends on STIM1 and ORAI1. Control and (A, G, H) STIM1-KO = STIM1^LysM-Cre+^, (B, I, J) ORAI1-KO = ORAI1^LysM-Cre+^, (C, K, L) STIM1/2-KO = STIM1/2^LysM-Cre+^, (D, M, N) STIM1/ORAI1-KO = STIM1/ORAI1^LysM-Cre+^, (E, O, P) STIM2-KO = STIM2^LysM-Cre+^, (F, Q, R) ORAI2-KO = ORAI2^LysM-Cre+^ and (S, T, U) control LysM^Cre+^ and ORAI1-KO = ORAI1^LysM-Cre+^ mice were injected intrascrotally with TNF-𝛼 (2h) and analyzed for leukocyte recruitment. Representative postcapillary cremaster muscle venules were analyzed for (A-F + S) neutrophil rolling velocity and (G, I, K, M, O, Q, T) the number of adherent neutrophils. (H, J, L, N, P, R, U) Leukocyte transmigration was visualized using reflected light oblique transillumination microscopy (RLOT) and examined for the number of emigrated cells, *n*=3, number of mice. (V) In control, LysM^cre+^ and ORAI1-KO = ORAI1^LysMcre+^ neutrophils, either unstimulated or stimulated with CXCL-1, binding of fluorescently coupled β_2_-integrin ligand ICAM-1 was assessed by flow cytometry, *n*=3, experimental repeat. Data are mean ± SEM. *p<0.05, **p<0.01, ***p<0.0001, ns=non significant by one-way ANOVA.

## Data Availability

All mouse strains and materials used in this study are available upon request. Please contact the lead contact. Immunoblot images, data, and microscopy images reported in this paper will be shared by the lead contact upon request.

## Abbreviations

AKI: Acute kidney injury
Ca^2+^: Calcium
CNS: Central nervous system
CRAC: Calcium-release activated calcium
DAG: Diacylglycerol
ER: Endoplasmic reticulum
GPCR: G-Protein coupled receptor
HBSS: Hanks balanced salt solution
ICAM-1: Intercellular adhesion molecule-1
IP_3_: Inositol triphosphate
Mac-1: Macrophage antigen-1
PGSL-1: P-selectin glycoprotein ligand-1
RLOT: Reflected light oblique transillumination
ROS: Reactive oxygen species
SICE: Store-independent calcium entry
SOCE: Store-operated calcium entry
STIM: Stromal interaction molecule
