# Supplementary material for: Integrin activation by two independently regulated calcium-mediated pathways is required for neutrophil recruitment
Source: Cell Commun Signal. 2026 Jan 21;24:57. doi: 10.1186/s12964-026-02666-w (PMC12849750; doi:10.1186/s12964-026-02666-w)
Supplement: Supplementary file 1 — Supplementary Material 1: Supplemental Fig. 1 Expression of Ca2+ isoforms in neutrophils. Determination of Ca2+ isoforms via immunoblotting from control and (A-B) STIM1-KO = STIM1LysM-Cre+, (C-D) ORAI1-KO =ORAI1LysM-Cre+, (E-G) STIM1/2-KO = STIM1/2LysM-Cre+, (H-J) STIM1/ORAI1-KO = STIM1/ORAI1LysM-Cre+, (K-L) STIM2-KO = STIM2LysM-Cre+ and (M-N) ORAI2-KO = ORAI2LysM-Cre+ neutrophils. Lysates were immunoblotted with (A, E, H) anti-STIM1, (C, H) anti-ORAI1, (E, K) anti-STIM2, (M) anti-ORAI2 and (A, C, E, H, K, M) anti-GAPDH, n=3, experimental repeat. Representative Western blot images are cropped. Data are mean ± SEM. *p<0.05, **p<0.01, ****p<0.0001 by one-way ANOVA. Supplemental Fig. 2. Calcium is required CXCL-1-induced CD11a activation. (A, B, C, F, G, H) Binding of fluorescently coupled β2-integrin ligands in unstimulated or CXCL-1 stimulated neutrophils. ICAM-1 binding to control neutrophils in presence of IgG or blocking anti-CD11a or anti-CD11b antibodies was assessed by flow cytometry, n=3, experimental repeat. In (B, C) control and Rap1a-KO = Rap1a-/- neutrophils binding of fluorescently coupled β2-integrin ligands (B) ICAM-1 and (C) fibrinogen was assessed by flow cytometry, n=3-4, experimental repeat. Intracellular calcium levels were analyzed in Fluo-4 labeled control neutrophils, incubated with either DMSO, (D) BAPTA or (E) thapsigargin before and after CXCL-1 stimulation. Binding of fluorescent coupled β2-integrin ligands (F, G) ICAM-1 and (H, I) fibrinogen to control neutrophils, incubated with either DMSO (control), (F, H) BAPTA or (G, I) thapsigargin was assessed by flow cytometry, n=3-5, experimental repeat. Data are mean ± SEM. *p<0.05, **p<0.01,***p<0.001, ****p<0.0001, ns=non significant by one-way ANOVA. Supplemental Fig. 3. Neutrophil recruitment depends on STIM1 and ORAI1. Control and (A, G, H) STIM1-KO = STIM1LysM-Cre+, (B, I, J) ORAI1-KO = ORAI1LysM-Cre+, (C, K, L) STIM1/2-KO = STIM1/2LysM-Cre+, (D, M, N) STIM1/ORAI1-KO = STIM1/ORAI1LysM-Cre+, [file 12964_2026_2666_MOESM1_ESM.zip › SupplementalFigure2_cell communication and signaling rev1(2).pptx]

## Slide 1
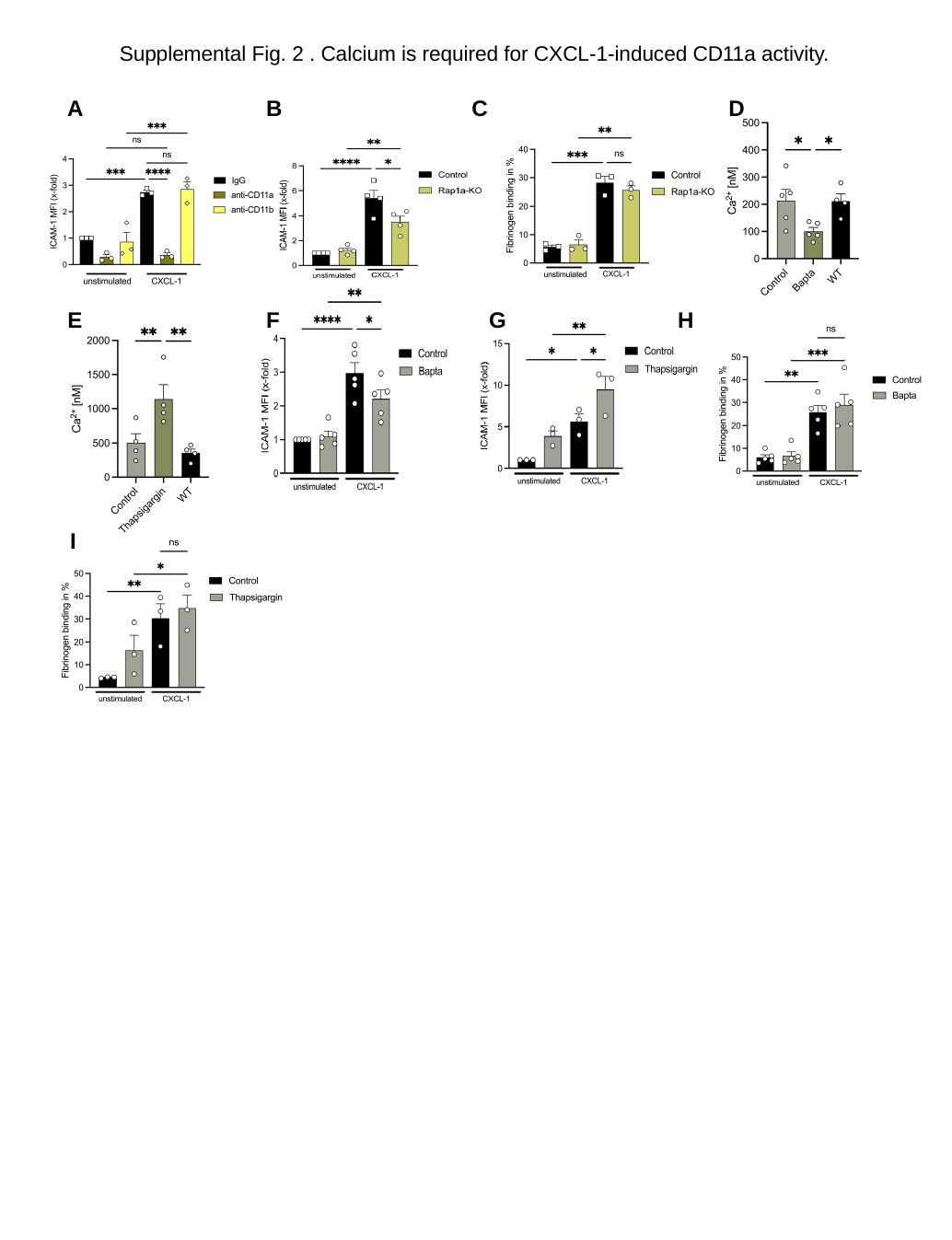

# Supplemental Fig. 2 . Calcium is required for CXCL-1-induced CD11a activity.
A
B
C
D
E
F
G
H
I

## Slide 2
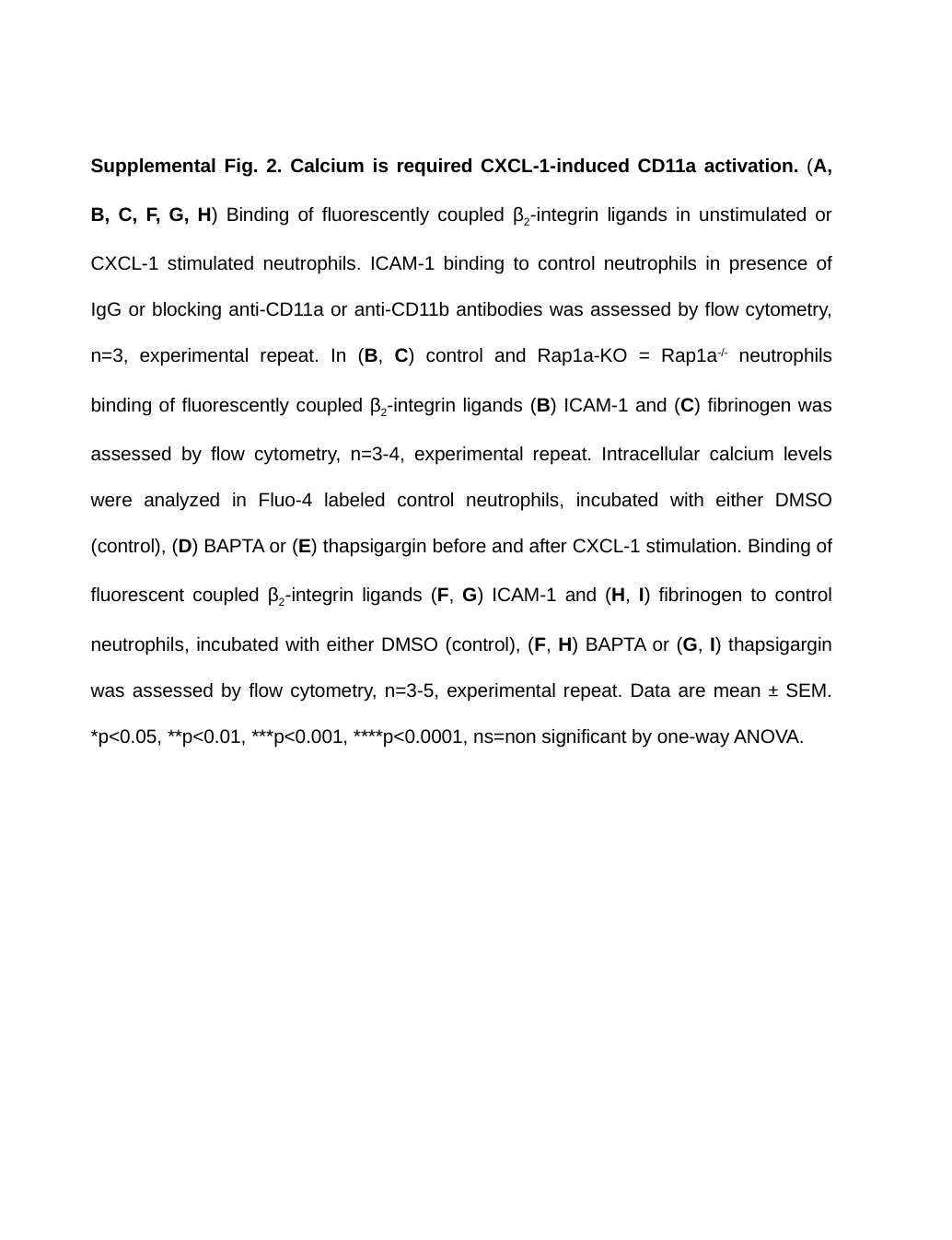

Supplemental Fig. 2. Calcium is required CXCL-1-induced CD11a activation. (A, B, C, F, G, H) Binding of fluorescently coupled β2-integrin ligands in unstimulated or CXCL-1 stimulated neutrophils. ICAM-1 binding to control neutrophils in presence of IgG or blocking anti-CD11a or anti-CD11b antibodies was assessed by flow cytometry, n=3, experimental repeat. In (B, C) control and Rap1a-KO = Rap1a-/- neutrophils binding of fluorescently coupled β2-integrin ligands (B) ICAM-1 and (C) fibrinogen was assessed by flow cytometry, n=3-4, experimental repeat. Intracellular calcium levels were analyzed in Fluo-4 labeled control neutrophils, incubated with either DMSO (control), (D) BAPTA or (E) thapsigargin before and after CXCL-1 stimulation. Binding of fluorescent coupled β2-integrin ligands (F, G) ICAM-1 and (H, I) fibrinogen to control neutrophils, incubated with either DMSO (control), (F, H) BAPTA or (G, I) thapsigargin was assessed by flow cytometry, n=3-5, experimental repeat. Data are mean ± SEM. *p<0.05, **p<0.01, ***p<0.001, ****p<0.0001, ns=non significant by one-way ANOVA.
