# Supplementary material for: Integrin activation by two independently regulated calcium-mediated pathways is required for neutrophil recruitment
Source: Cell Commun Signal. 2026 Jan 21;24:57. doi: 10.1186/s12964-026-02666-w (PMC12849750; doi:10.1186/s12964-026-02666-w)
Supplement: Supplementary file 1 — Supplementary Material 1: Supplemental Fig. 1 Expression of Ca2+ isoforms in neutrophils. Determination of Ca2+ isoforms via immunoblotting from control and (A-B) STIM1-KO = STIM1LysM-Cre+, (C-D) ORAI1-KO =ORAI1LysM-Cre+, (E-G) STIM1/2-KO = STIM1/2LysM-Cre+, (H-J) STIM1/ORAI1-KO = STIM1/ORAI1LysM-Cre+, (K-L) STIM2-KO = STIM2LysM-Cre+ and (M-N) ORAI2-KO = ORAI2LysM-Cre+ neutrophils. Lysates were immunoblotted with (A, E, H) anti-STIM1, (C, H) anti-ORAI1, (E, K) anti-STIM2, (M) anti-ORAI2 and (A, C, E, H, K, M) anti-GAPDH, n=3, experimental repeat. Representative Western blot images are cropped. Data are mean ± SEM. *p<0.05, **p<0.01, ****p<0.0001 by one-way ANOVA. Supplemental Fig. 2. Calcium is required CXCL-1-induced CD11a activation. (A, B, C, F, G, H) Binding of fluorescently coupled β2-integrin ligands in unstimulated or CXCL-1 stimulated neutrophils. ICAM-1 binding to control neutrophils in presence of IgG or blocking anti-CD11a or anti-CD11b antibodies was assessed by flow cytometry, n=3, experimental repeat. In (B, C) control and Rap1a-KO = Rap1a-/- neutrophils binding of fluorescently coupled β2-integrin ligands (B) ICAM-1 and (C) fibrinogen was assessed by flow cytometry, n=3-4, experimental repeat. Intracellular calcium levels were analyzed in Fluo-4 labeled control neutrophils, incubated with either DMSO, (D) BAPTA or (E) thapsigargin before and after CXCL-1 stimulation. Binding of fluorescent coupled β2-integrin ligands (F, G) ICAM-1 and (H, I) fibrinogen to control neutrophils, incubated with either DMSO (control), (F, H) BAPTA or (G, I) thapsigargin was assessed by flow cytometry, n=3-5, experimental repeat. Data are mean ± SEM. *p<0.05, **p<0.01,***p<0.001, ****p<0.0001, ns=non significant by one-way ANOVA. Supplemental Fig. 3. Neutrophil recruitment depends on STIM1 and ORAI1. Control and (A, G, H) STIM1-KO = STIM1LysM-Cre+, (B, I, J) ORAI1-KO = ORAI1LysM-Cre+, (C, K, L) STIM1/2-KO = STIM1/2LysM-Cre+, (D, M, N) STIM1/ORAI1-KO = STIM1/ORAI1LysM-Cre+, [file 12964_2026_2666_MOESM1_ESM.zip › SupplementalFigure3_cell communication and signaling rev1(3).pptx]

## Slide 1
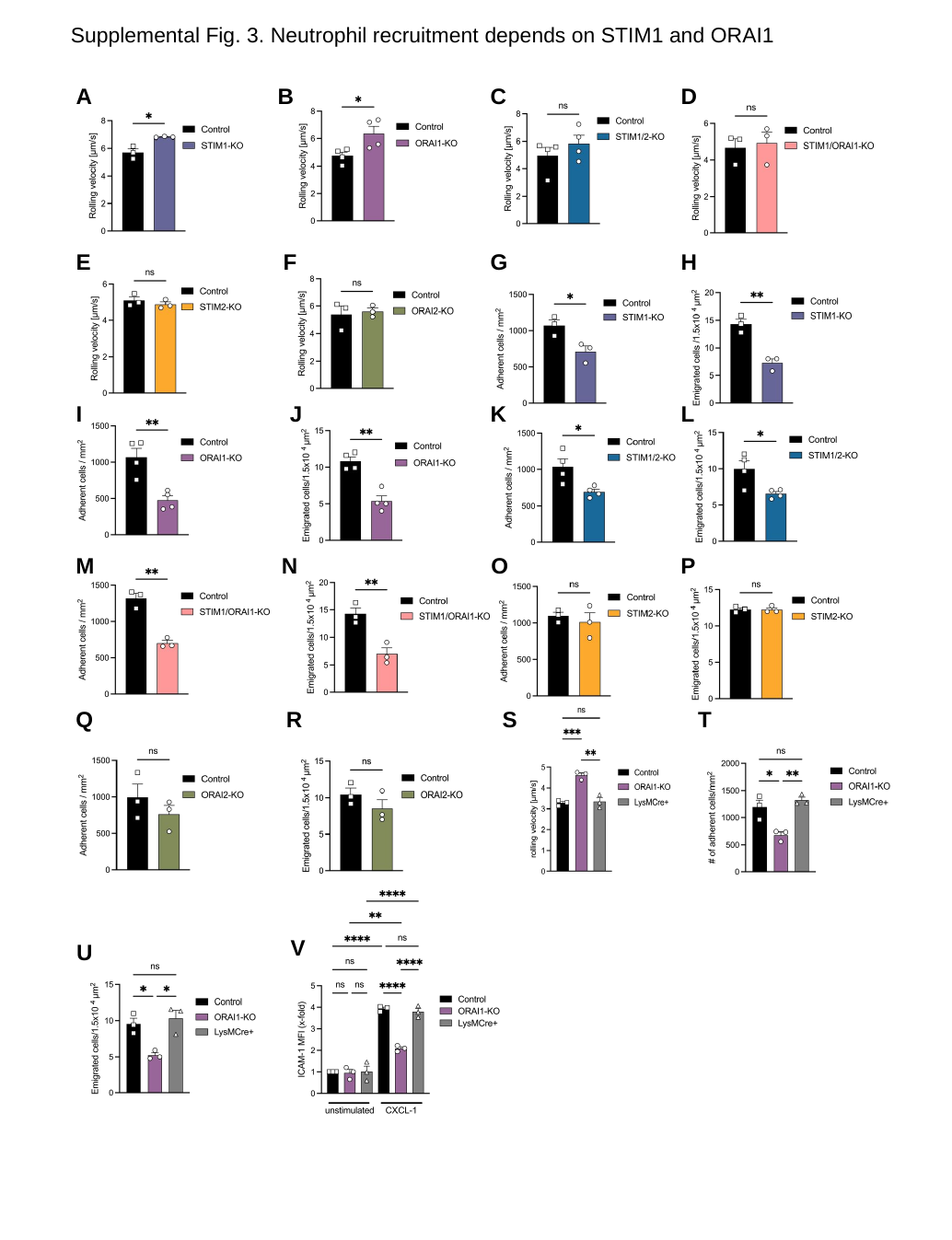

# Supplemental Fig. 3. Neutrophil recruitment depends on STIM1 and ORAI1
A
B
C
D
E
F
G
H
I
J
K
L
M
N
O
P
Q
R
S
T
V
U

## Slide 2
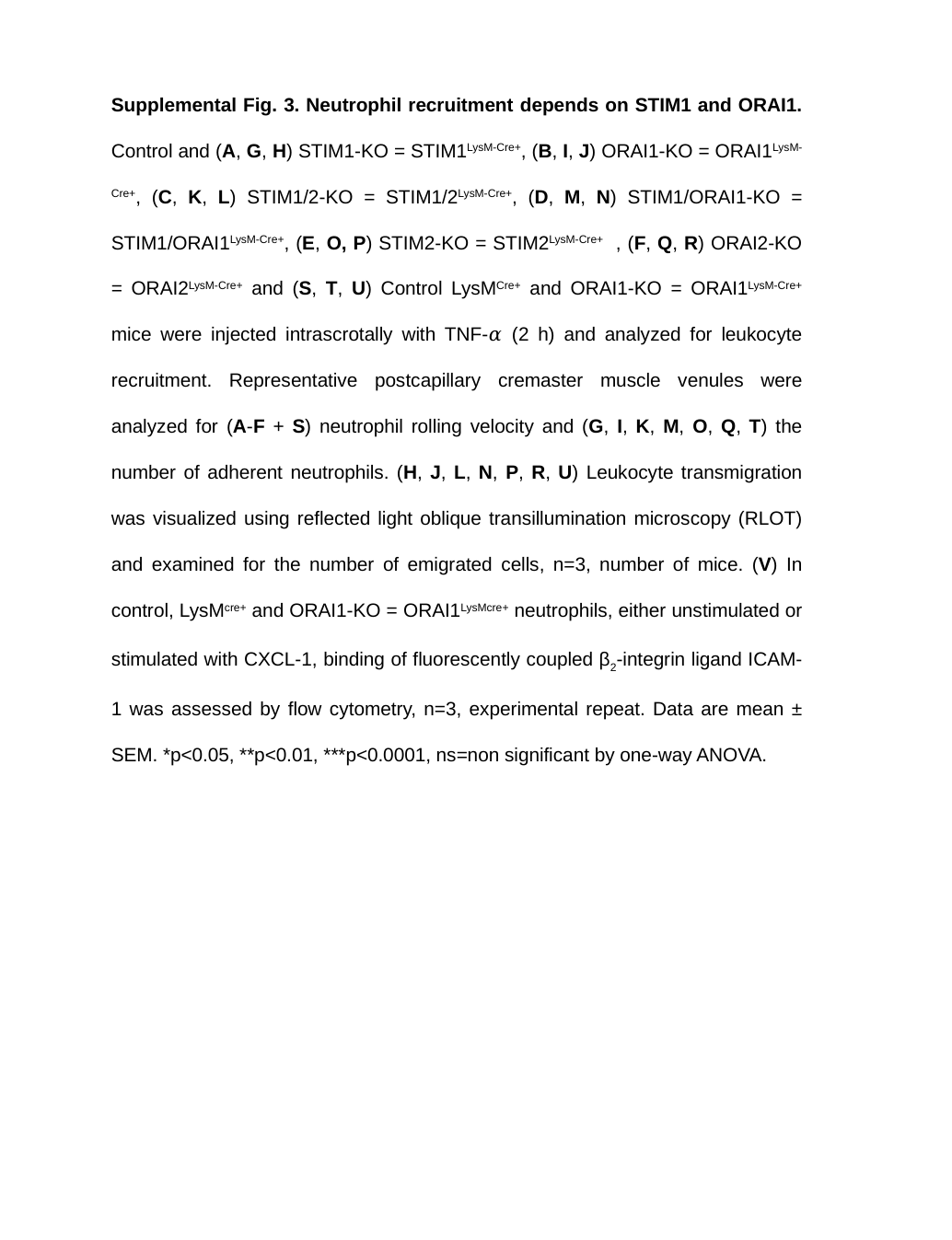

Supplemental Fig. 3. Neutrophil recruitment depends on STIM1 and ORAI1. Control and (A, G, H) STIM1-KO = STIM1LysM-Cre+, (B, I, J) ORAI1-KO = ORAI1LysM-Cre+, (C, K, L) STIM1/2-KO = STIM1/2LysM-Cre+, (D, M, N) STIM1/ORAI1-KO = STIM1/ORAI1LysM-Cre+, (E, O, P) STIM2-KO = STIM2LysM-Cre+ , (F, Q, R) ORAI2-KO = ORAI2LysM-Cre+ and (S, T, U) Control LysMCre+ and ORAI1-KO = ORAI1LysM-Cre+ mice were injected intrascrotally with TNF-𝛼 (2 h) and analyzed for leukocyte recruitment. Representative postcapillary cremaster muscle venules were analyzed for (A-F + S) neutrophil rolling velocity and (G, I, K, M, O, Q, T) the number of adherent neutrophils. (H, J, L, N, P, R, U) Leukocyte transmigration was visualized using reflected light oblique transillumination microscopy (RLOT) and examined for the number of emigrated cells, n=3, number of mice. (V) In control, LysMcre+ and ORAI1-KO = ORAI1LysMcre+ neutrophils, either unstimulated or stimulated with CXCL-1, binding of fluorescently coupled β2-integrin ligand ICAM-1 was assessed by flow cytometry, n=3, experimental repeat. Data are mean ± SEM. *p<0.05, **p<0.01, ***p<0.0001, ns=non significant by one-way ANOVA.
