# Supplementary material for: Integrin activation by two independently regulated calcium-mediated pathways is required for neutrophil recruitment
Source: Cell Commun Signal. 2026 Jan 21;24:57. doi: 10.1186/s12964-026-02666-w (PMC12849750; doi:10.1186/s12964-026-02666-w)
Supplement: Supplementary file 1 — Supplementary Material 1: Supplemental Fig. 1 Expression of Ca2+ isoforms in neutrophils. Determination of Ca2+ isoforms via immunoblotting from control and (A-B) STIM1-KO = STIM1LysM-Cre+, (C-D) ORAI1-KO =ORAI1LysM-Cre+, (E-G) STIM1/2-KO = STIM1/2LysM-Cre+, (H-J) STIM1/ORAI1-KO = STIM1/ORAI1LysM-Cre+, (K-L) STIM2-KO = STIM2LysM-Cre+ and (M-N) ORAI2-KO = ORAI2LysM-Cre+ neutrophils. Lysates were immunoblotted with (A, E, H) anti-STIM1, (C, H) anti-ORAI1, (E, K) anti-STIM2, (M) anti-ORAI2 and (A, C, E, H, K, M) anti-GAPDH, n=3, experimental repeat. Representative Western blot images are cropped. Data are mean ± SEM. *p<0.05, **p<0.01, ****p<0.0001 by one-way ANOVA. Supplemental Fig. 2. Calcium is required CXCL-1-induced CD11a activation. (A, B, C, F, G, H) Binding of fluorescently coupled β2-integrin ligands in unstimulated or CXCL-1 stimulated neutrophils. ICAM-1 binding to control neutrophils in presence of IgG or blocking anti-CD11a or anti-CD11b antibodies was assessed by flow cytometry, n=3, experimental repeat. In (B, C) control and Rap1a-KO = Rap1a-/- neutrophils binding of fluorescently coupled β2-integrin ligands (B) ICAM-1 and (C) fibrinogen was assessed by flow cytometry, n=3-4, experimental repeat. Intracellular calcium levels were analyzed in Fluo-4 labeled control neutrophils, incubated with either DMSO, (D) BAPTA or (E) thapsigargin before and after CXCL-1 stimulation. Binding of fluorescent coupled β2-integrin ligands (F, G) ICAM-1 and (H, I) fibrinogen to control neutrophils, incubated with either DMSO (control), (F, H) BAPTA or (G, I) thapsigargin was assessed by flow cytometry, n=3-5, experimental repeat. Data are mean ± SEM. *p<0.05, **p<0.01,***p<0.001, ****p<0.0001, ns=non significant by one-way ANOVA. Supplemental Fig. 3. Neutrophil recruitment depends on STIM1 and ORAI1. Control and (A, G, H) STIM1-KO = STIM1LysM-Cre+, (B, I, J) ORAI1-KO = ORAI1LysM-Cre+, (C, K, L) STIM1/2-KO = STIM1/2LysM-Cre+, (D, M, N) STIM1/ORAI1-KO = STIM1/ORAI1LysM-Cre+, [file 12964_2026_2666_MOESM1_ESM.zip › supplementary file_cell communication and signaling rev1.pptx]

## Slide 1
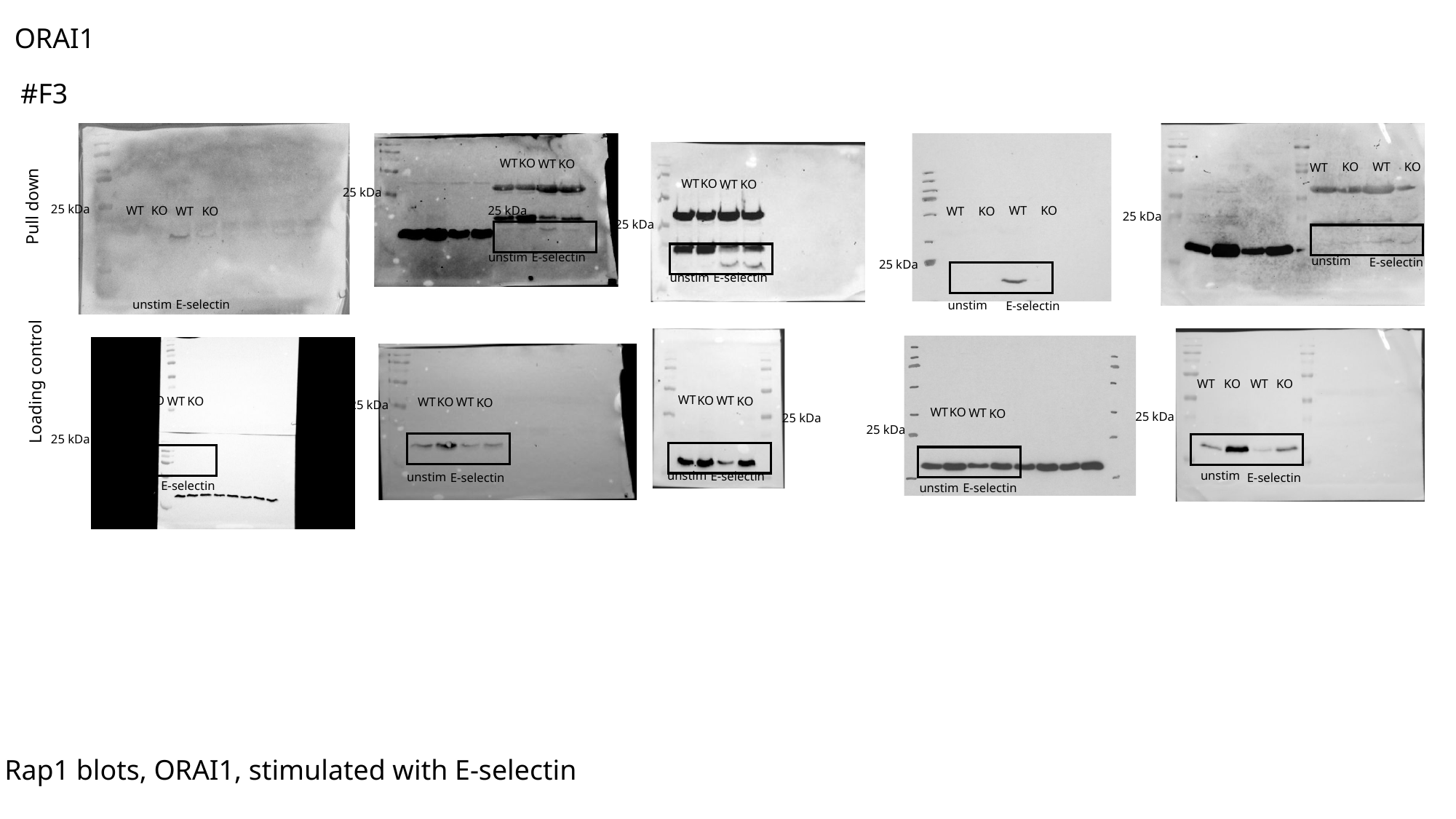

ORAI1
#F3
WT
KO
WT
KO
WT
KO
KO
WT
WT
KO
WT
KO
25 kDa
Pull down
25 kDa
25 kDa
WT
WT
KO
KO
KO
WT
KO
WT
25 kDa
25 kDa
unstim
E-selectin
unstim
E-selectin
25 kDa
unstim
E-selectin
unstim
E-selectin
unstim
E-selectin
Loading control
WT
KO
WT
KO
WT
KO
WT
WT
KO
KO
WT
KO
WT
KO
WT
KO
25 kDa
WT
KO
WT
KO
25 kDa
25 kDa
25 kDa
25 kDa
unstim
unstim
E-selectin
unstim
E-selectin
E-selectin
unstim
E-selectin
unstim
E-selectin
Rap1 blots, ORAI1, stimulated with E-selectin

## Slide 2
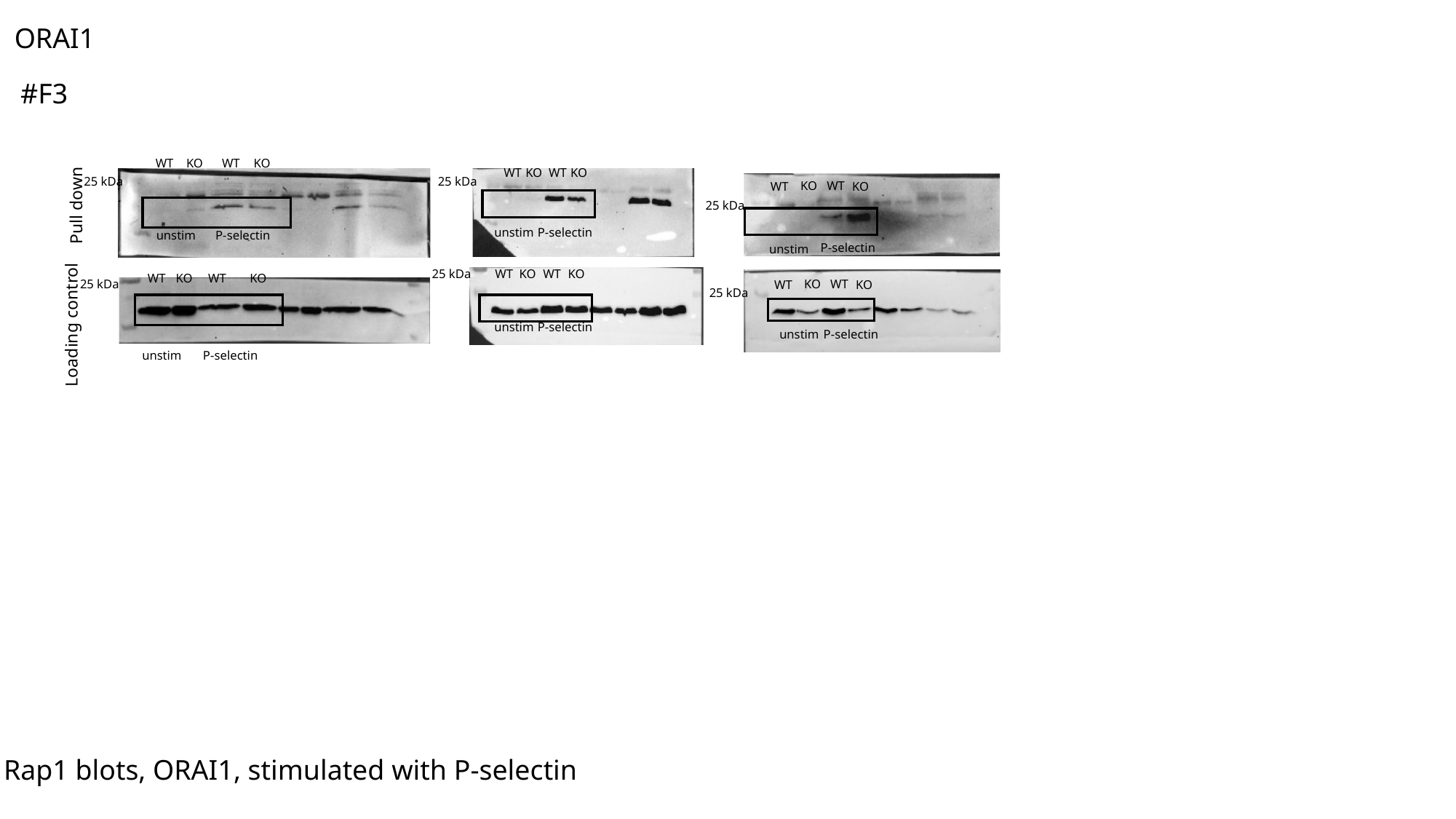

ORAI1
#F3
WT
KO
WT
KO
WT
KO
WT
KO
25 kDa
25 kDa
KO
WT
WT
KO
Pull down
25 kDa
unstim
P-selectin
unstim
P-selectin
P-selectin
unstim
KO
WT
KO
25 kDa
WT
WT
KO
WT
KO
KO
WT
25 kDa
WT
KO
25 kDa
Loading control
unstim
P-selectin
unstim
P-selectin
unstim
P-selectin
Rap1 blots, ORAI1, stimulated with P-selectin

## Slide 3
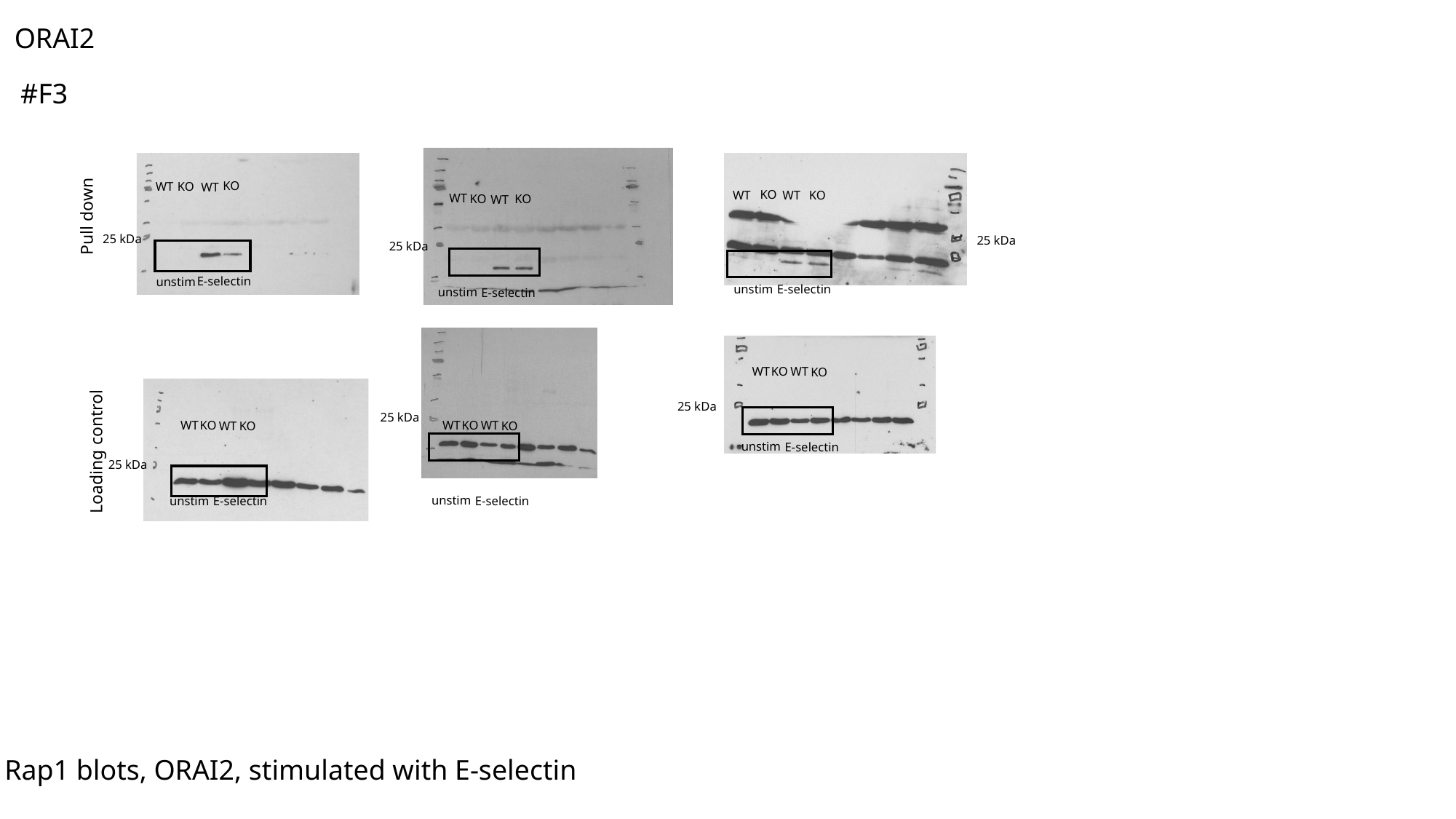

ORAI2
#F3
KO
WT
KO
WT
KO
WT
WT
KO
WT
KO
KO
WT
Pull down
25 kDa
25 kDa
25 kDa
E-selectin
unstim
unstim
E-selectin
unstim
E-selectin
WT
KO
WT
KO
25 kDa
25 kDa
WT
KO
WT
KO
WT
WT
KO
KO
unstim
E-selectin
Loading control
25 kDa
unstim
unstim
E-selectin
E-selectin
Rap1 blots, ORAI2, stimulated with E-selectin

## Slide 4
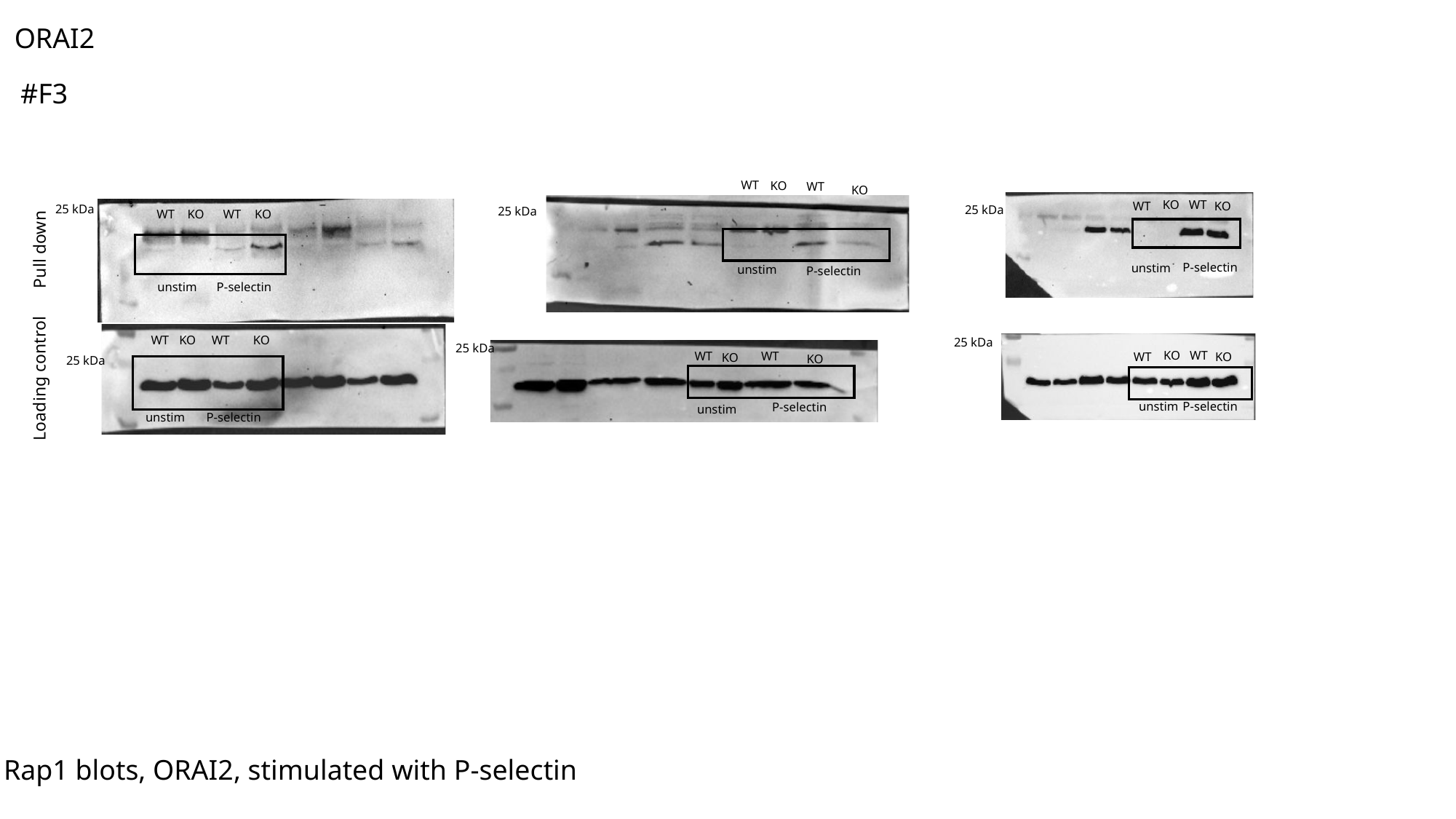

ORAI2
#F3
WT
KO
WT
KO
KO
WT
WT
KO
25 kDa
25 kDa
25 kDa
WT
KO
WT
KO
Pull down
P-selectin
unstim
unstim
P-selectin
unstim
P-selectin
WT
KO
WT
KO
25 kDa
25 kDa
KO
WT
WT
WT
WT
KO
KO
KO
25 kDa
Loading control
unstim
P-selectin
P-selectin
unstim
unstim
P-selectin
Rap1 blots, ORAI2, stimulated with P-selectin

## Slide 5
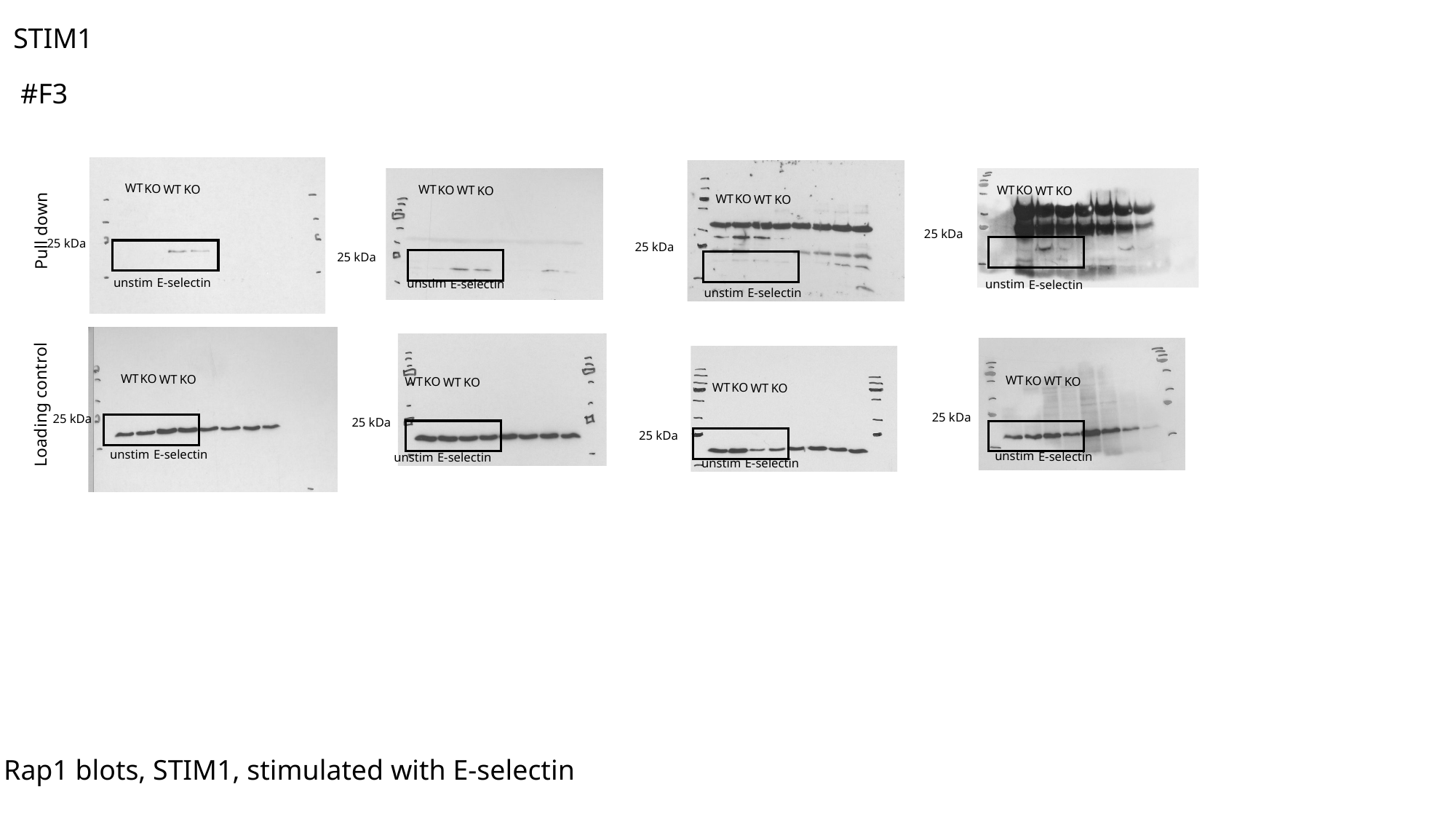

STIM1
#F3
WT
KO
WT
KO
WT
KO
WT
WT
KO
KO
WT
KO
WT
KO
WT
KO
Pull down
25 kDa
25 kDa
25 kDa
25 kDa
unstim
E-selectin
unstim
E-selectin
unstim
E-selectin
unstim
E-selectin
WT
KO
WT
KO
WT
KO
WT
KO
WT
KO
WT
KO
WT
KO
WT
KO
Loading control
25 kDa
25 kDa
25 kDa
25 kDa
unstim
E-selectin
unstim
E-selectin
unstim
E-selectin
unstim
E-selectin
Rap1 blots, STIM1, stimulated with E-selectin

## Slide 6
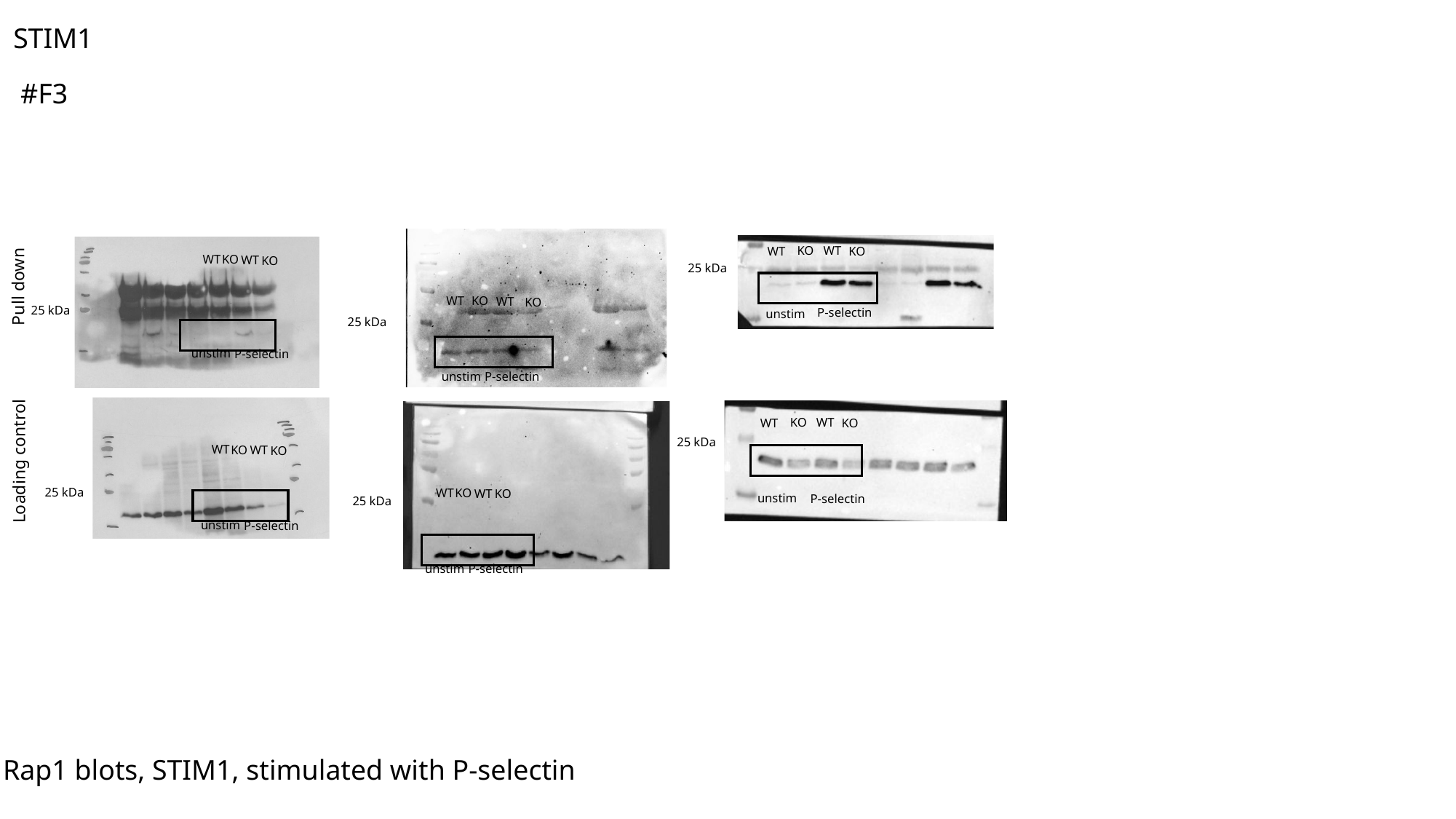

STIM1
#F3
KO
WT
WT
KO
WT
KO
WT
KO
25 kDa
Pull down
WT
KO
WT
KO
25 kDa
P-selectin
unstim
25 kDa
unstim
P-selectin
unstim
P-selectin
KO
WT
WT
KO
25 kDa
WT
KO
WT
KO
Loading control
25 kDa
WT
KO
WT
KO
unstim
P-selectin
25 kDa
unstim
P-selectin
unstim
P-selectin
Rap1 blots, STIM1, stimulated with P-selectin

## Slide 7
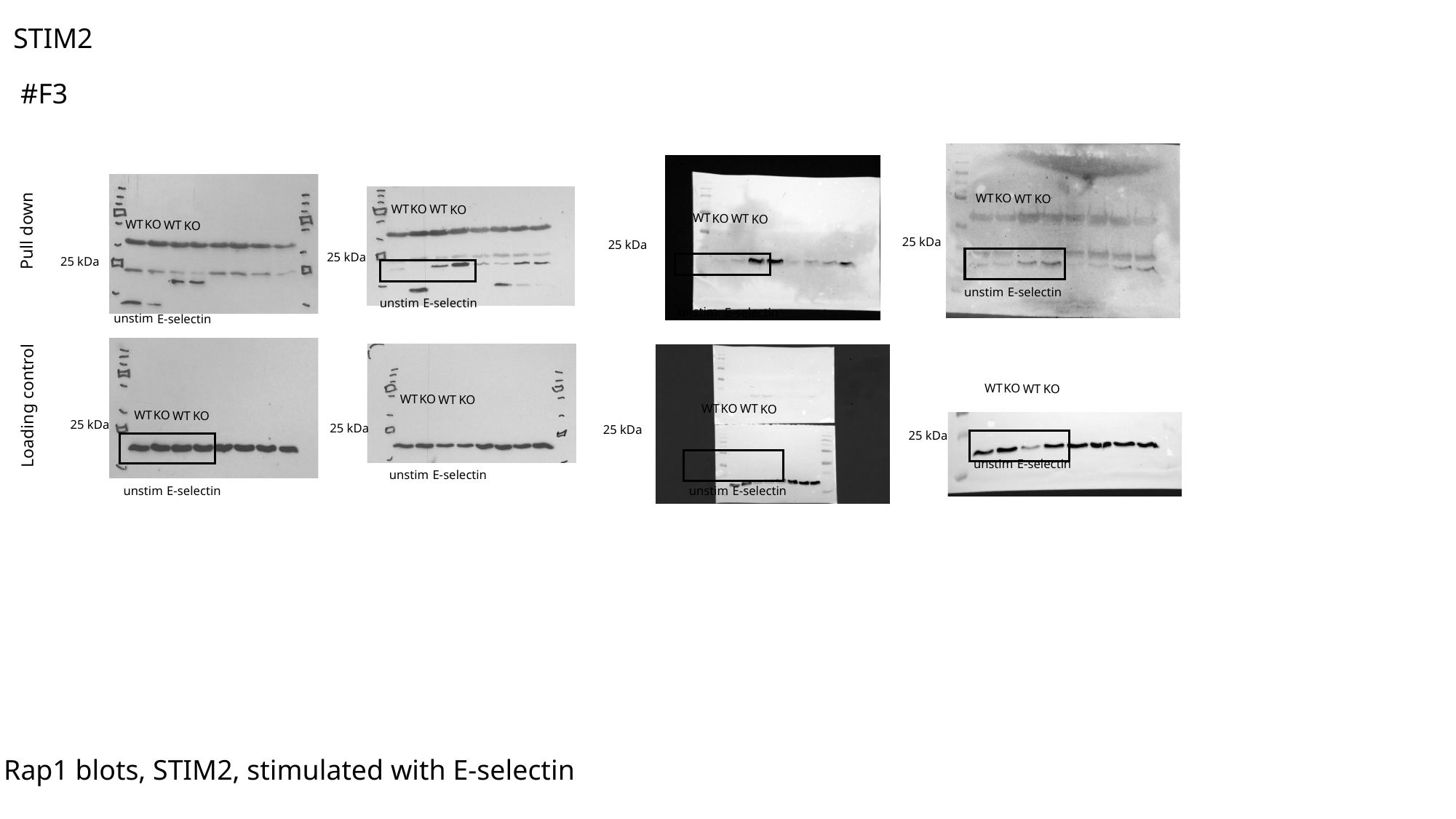

STIM2
#F3
WT
KO
WT
KO
WT
KO
WT
KO
WT
KO
WT
KO
WT
KO
WT
KO
Pull down
25 kDa
25 kDa
25 kDa
25 kDa
unstim
E-selectin
unstim
E-selectin
unstim
E-selectin
unstim
E-selectin
WT
KO
WT
KO
WT
KO
WT
KO
Loading control
WT
KO
WT
KO
WT
KO
WT
KO
25 kDa
25 kDa
25 kDa
25 kDa
unstim
E-selectin
unstim
E-selectin
unstim
unstim
E-selectin
E-selectin
Rap1 blots, STIM2, stimulated with E-selectin

## Slide 8
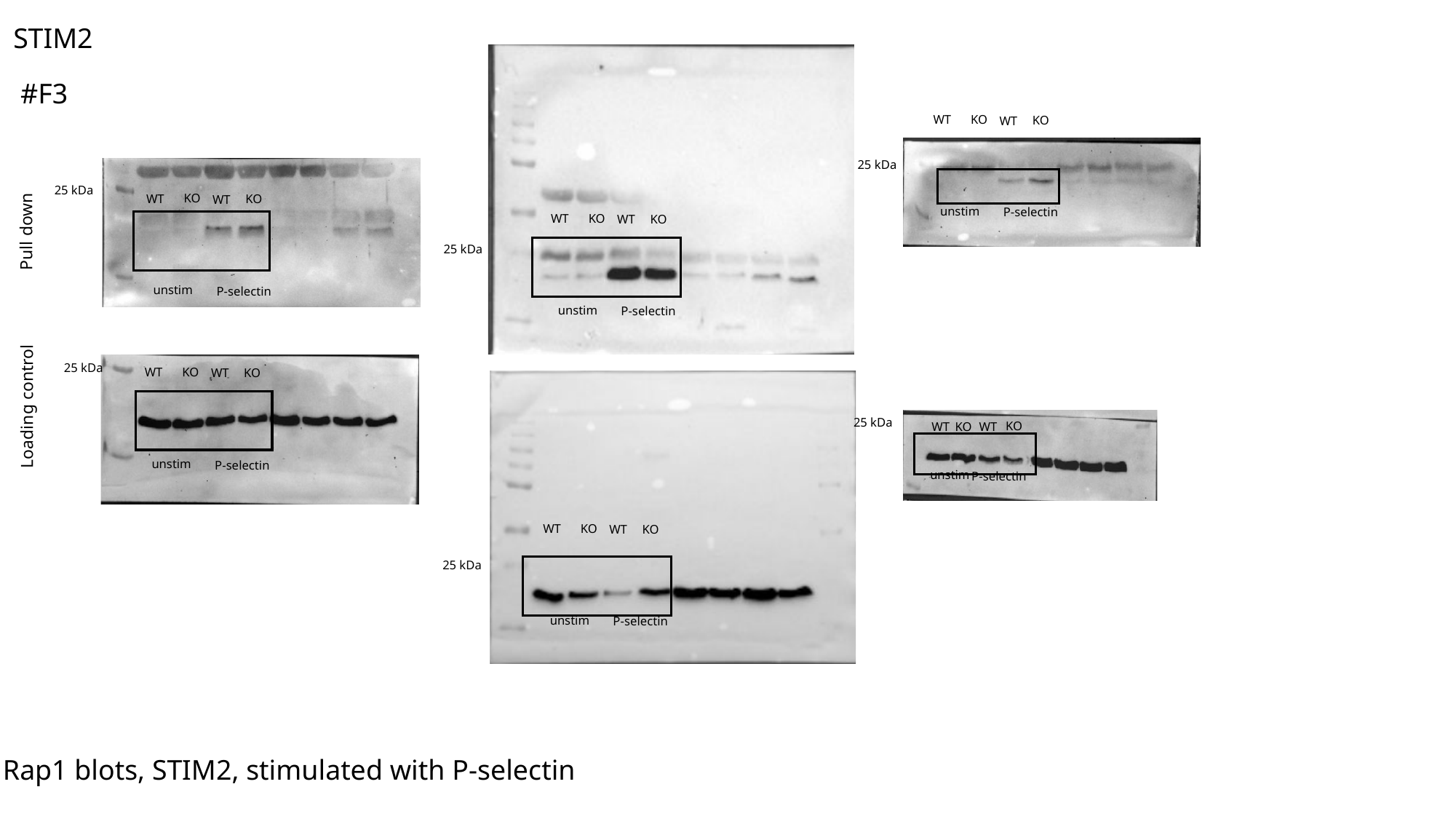

STIM2
#F3
KO
WT
KO
WT
25 kDa
25 kDa
KO
WT
KO
WT
unstim
P-selectin
KO
WT
KO
WT
Pull down
25 kDa
unstim
P-selectin
unstim
P-selectin
25 kDa
KO
WT
KO
WT
Loading control
25 kDa
KO
KO
WT
WT
unstim
P-selectin
unstim
P-selectin
KO
WT
KO
WT
25 kDa
unstim
P-selectin
Rap1 blots, STIM2, stimulated with P-selectin

## Slide 9
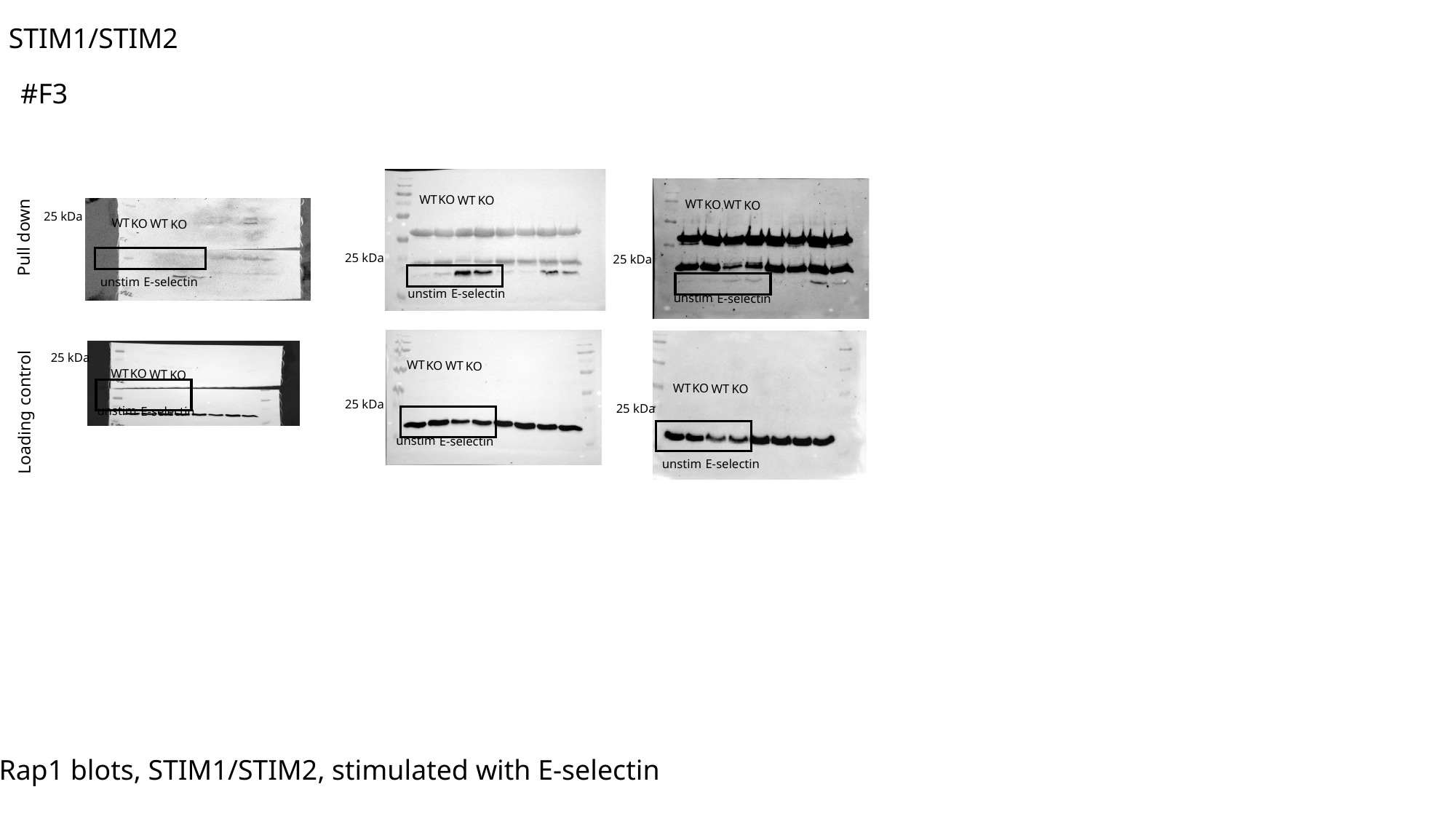

STIM1/STIM2
#F3
WT
KO
WT
KO
WT
KO
WT
KO
25 kDa
WT
KO
WT
KO
Pull down
25 kDa
25 kDa
unstim
E-selectin
unstim
E-selectin
unstim
E-selectin
25 kDa
WT
KO
WT
KO
WT
KO
WT
KO
WT
KO
WT
KO
25 kDa
25 kDa
Loading control
unstim
E-selectin
unstim
E-selectin
unstim
E-selectin
Rap1 blots, STIM1/STIM2, stimulated with E-selectin

## Slide 10
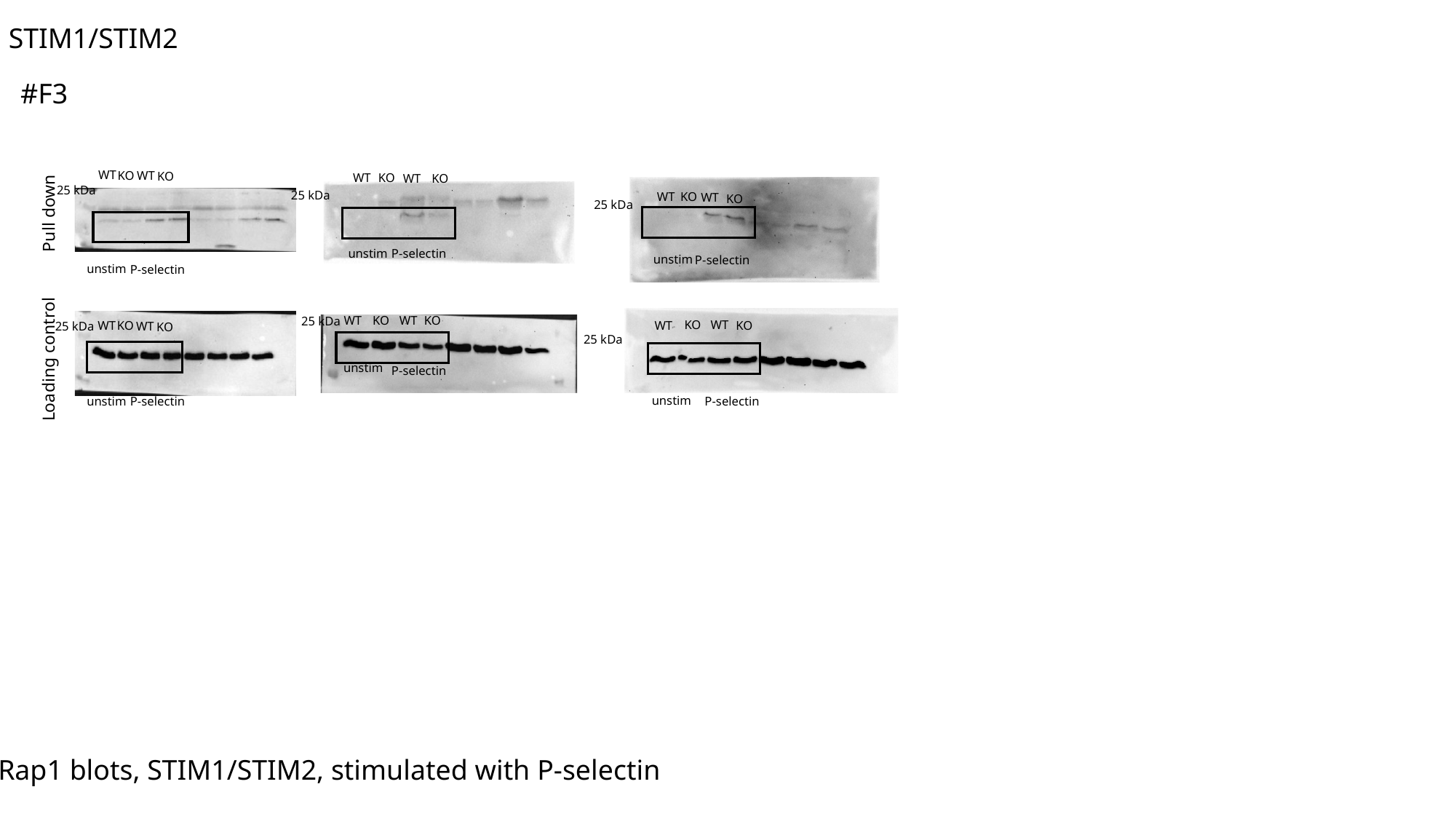

STIM1/STIM2
#F3
WT
KO
WT
KO
WT
KO
WT
KO
25 kDa
25 kDa
WT
KO
WT
KO
25 kDa
Pull down
unstim
P-selectin
unstim
P-selectin
unstim
P-selectin
KO
KO
WT
WT
25 kDa
KO
WT
WT
WT
KO
KO
WT
25 kDa
KO
25 kDa
Loading control
unstim
P-selectin
unstim
unstim
P-selectin
P-selectin
Rap1 blots, STIM1/STIM2, stimulated with P-selectin

## Slide 11
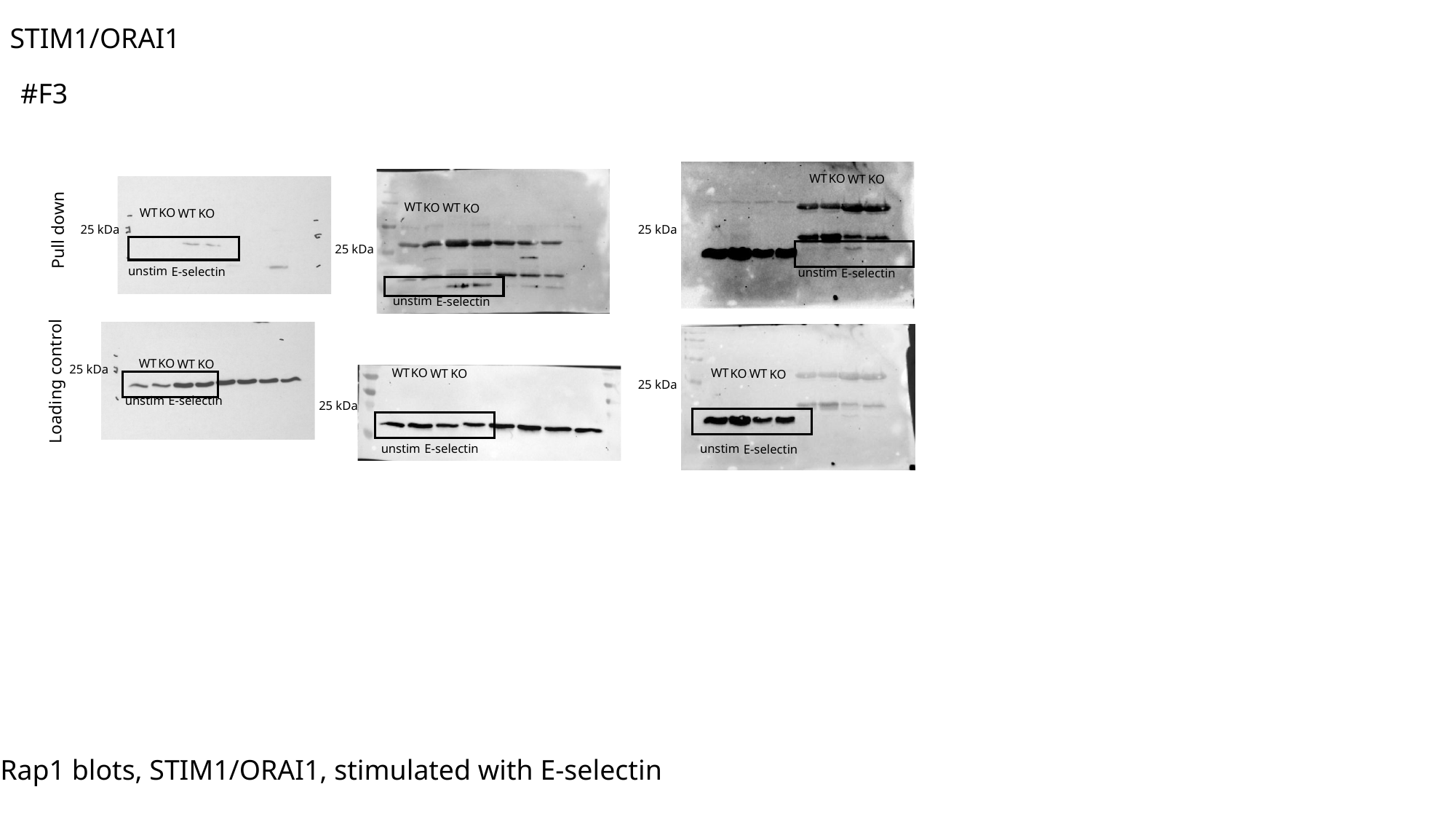

STIM1/ORAI1
#F3
WT
KO
WT
KO
WT
KO
WT
KO
WT
KO
WT
KO
Pull down
25 kDa
25 kDa
25 kDa
unstim
E-selectin
unstim
E-selectin
unstim
E-selectin
WT
KO
WT
KO
25 kDa
WT
KO
WT
KO
WT
KO
WT
KO
Loading control
25 kDa
unstim
E-selectin
25 kDa
unstim
E-selectin
unstim
E-selectin
Rap1 blots, STIM1/ORAI1, stimulated with E-selectin

## Slide 12
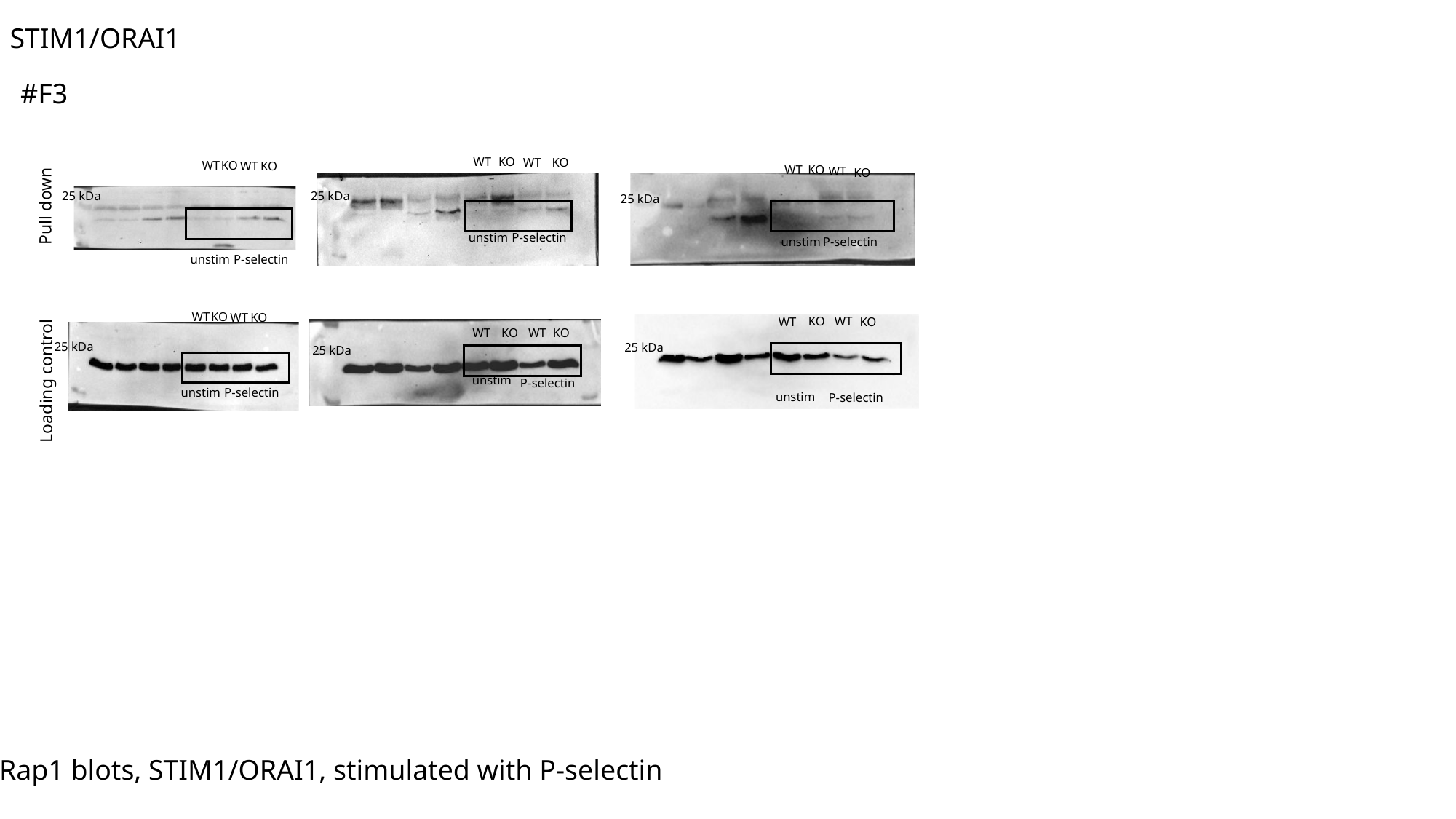

STIM1/ORAI1
#F3
WT
KO
WT
KO
WT
KO
WT
KO
WT
KO
WT
KO
25 kDa
25 kDa
25 kDa
Pull down
unstim
P-selectin
unstim
P-selectin
unstim
P-selectin
WT
KO
WT
KO
KO
WT
WT
KO
KO
KO
WT
WT
25 kDa
25 kDa
25 kDa
Loading control
unstim
P-selectin
unstim
P-selectin
unstim
P-selectin
Rap1 blots, STIM1/ORAI1, stimulated with P-selectin

## Slide 13
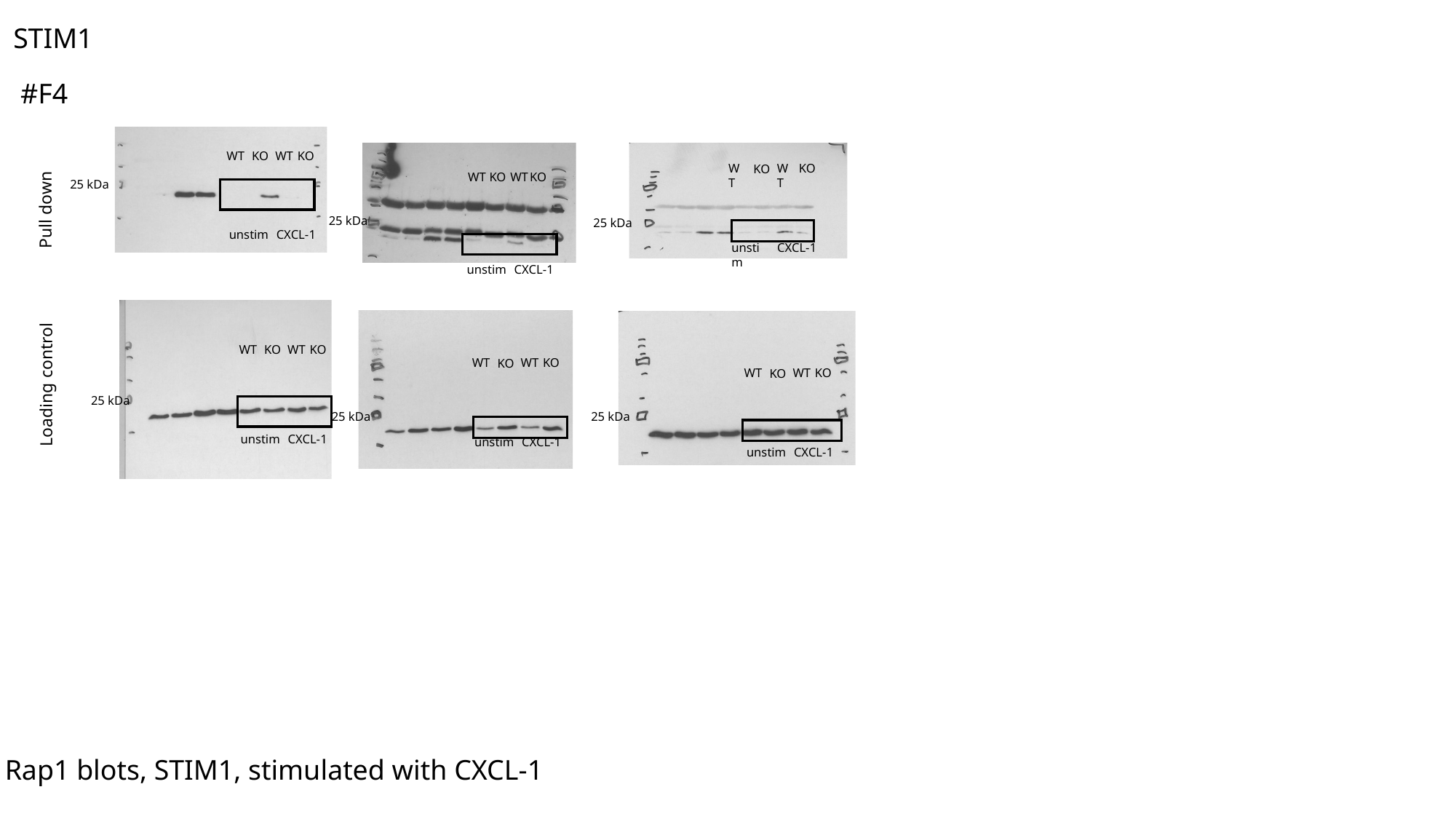

STIM1
#F4
WT
WT
KO
KO
WT
WT
KO
KO
WT
KO
WT
KO
25 kDa
Pull down
25 kDa
25 kDa
unstim
CXCL-1
unstim
CXCL-1
unstim
CXCL-1
WT
WT
KO
KO
WT
WT
KO
KO
WT
WT
KO
KO
Loading control
25 kDa
25 kDa
25 kDa
unstim
CXCL-1
unstim
CXCL-1
unstim
CXCL-1
Rap1 blots, STIM1, stimulated with CXCL-1

## Slide 14
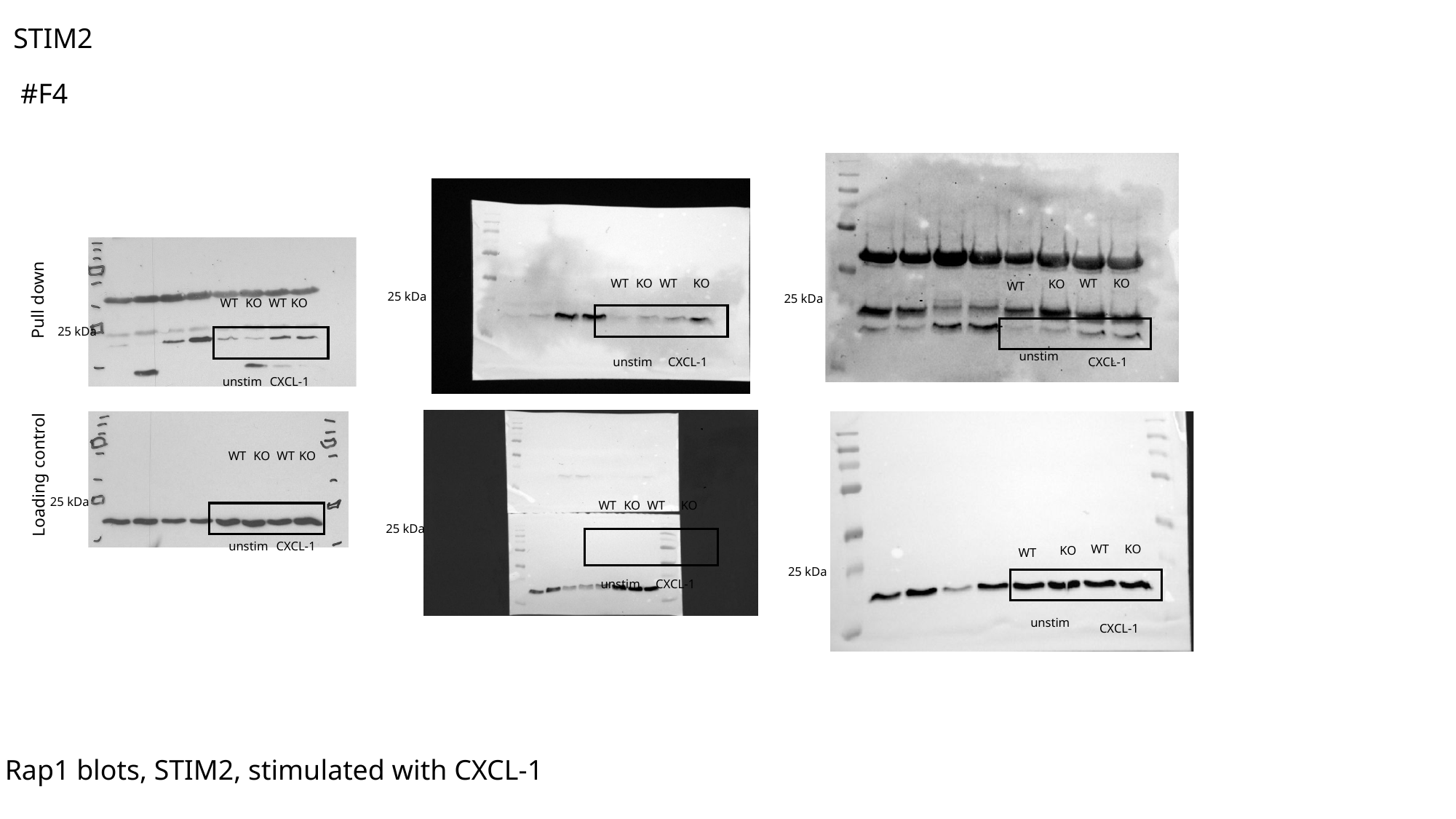

STIM2
#F4
WT
WT
KO
WT
KO
KO
KO
WT
Pull down
25 kDa
25 kDa
WT
WT
KO
KO
25 kDa
unstim
unstim
CXCL-1
CXCL-1
unstim
CXCL-1
WT
WT
KO
KO
Loading control
25 kDa
WT
WT
KO
KO
25 kDa
unstim
CXCL-1
WT
KO
KO
WT
25 kDa
unstim
CXCL-1
unstim
CXCL-1
Rap1 blots, STIM2, stimulated with CXCL-1

## Slide 15
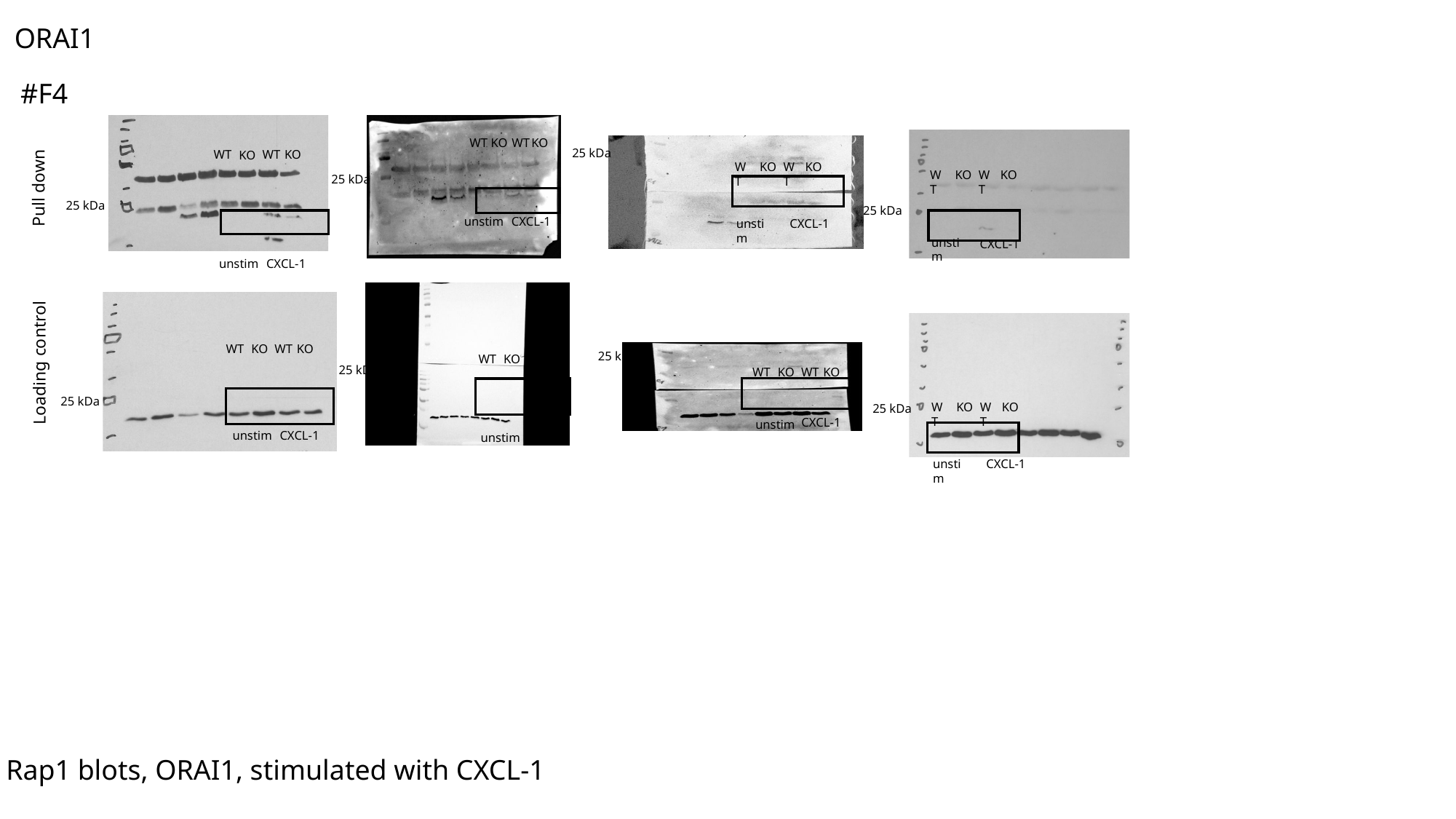

ORAI1
#F4
WT
KO
WT
KO
25 kDa
WT
WT
KO
KO
WT
WT
KO
KO
WT
WT
KO
KO
25 kDa
Pull down
25 kDa
25 kDa
unstim
CXCL-1
unstim
CXCL-1
unstim
CXCL-1
unstim
CXCL-1
WT
WT
KO
KO
25 kDa
WT
WT
KO
KO
Loading control
25 kDa
WT
WT
KO
KO
25 kDa
WT
WT
KO
KO
25 kDa
CXCL-1
unstim
unstim
CXCL-1
unstim
CXCL-1
unstim
CXCL-1
Rap1 blots, ORAI1, stimulated with CXCL-1

## Slide 16
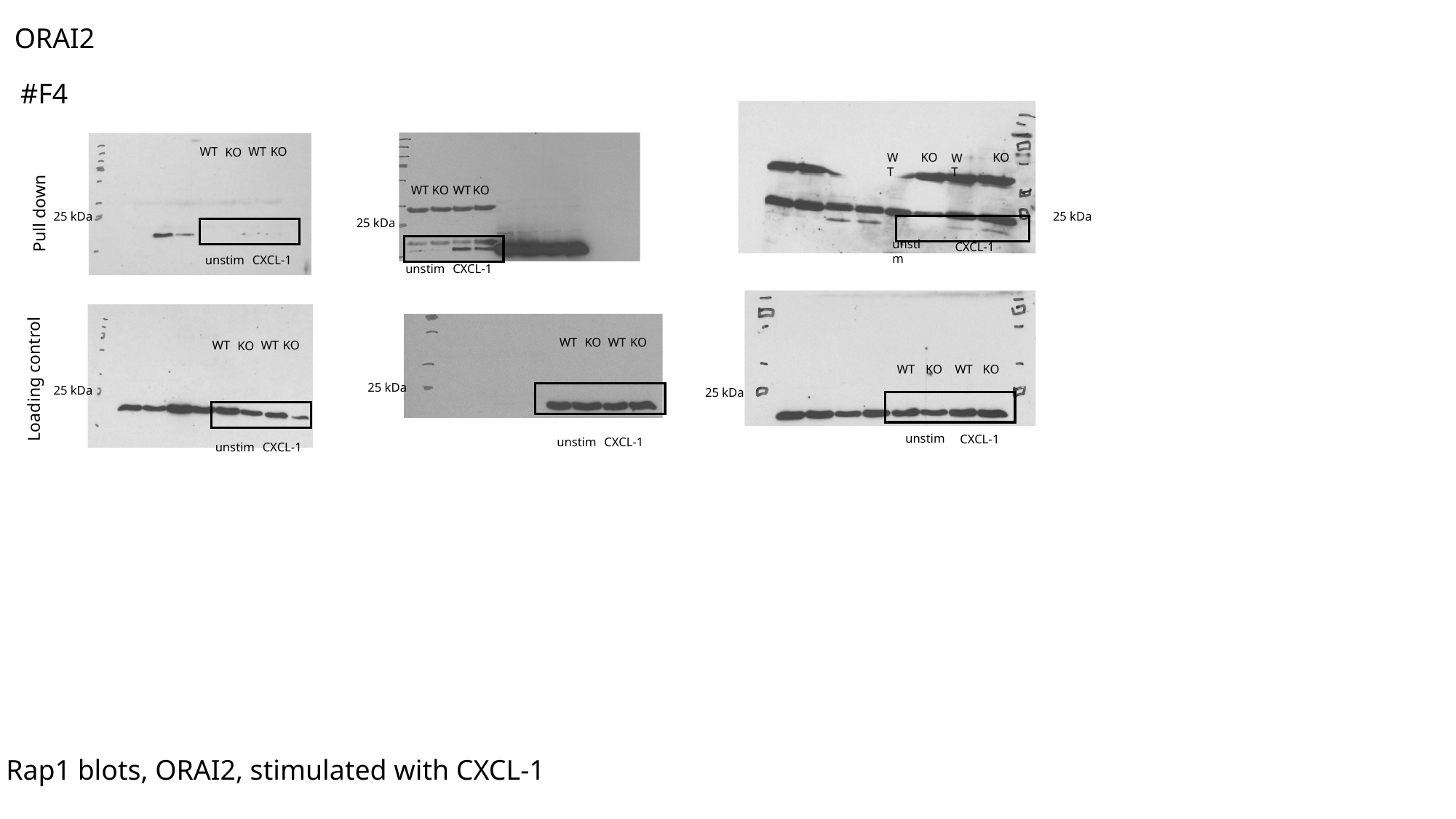

ORAI2
#F4
WT
WT
KO
KO
WT
KO
KO
WT
WT
KO
WT
KO
Pull down
25 kDa
25 kDa
25 kDa
unstim
CXCL-1
unstim
CXCL-1
unstim
CXCL-1
WT
WT
KO
KO
WT
WT
KO
KO
WT
KO
WT
KO
Loading control
25 kDa
25 kDa
25 kDa
unstim
CXCL-1
unstim
CXCL-1
unstim
CXCL-1
Rap1 blots, ORAI2, stimulated with CXCL-1

## Slide 17
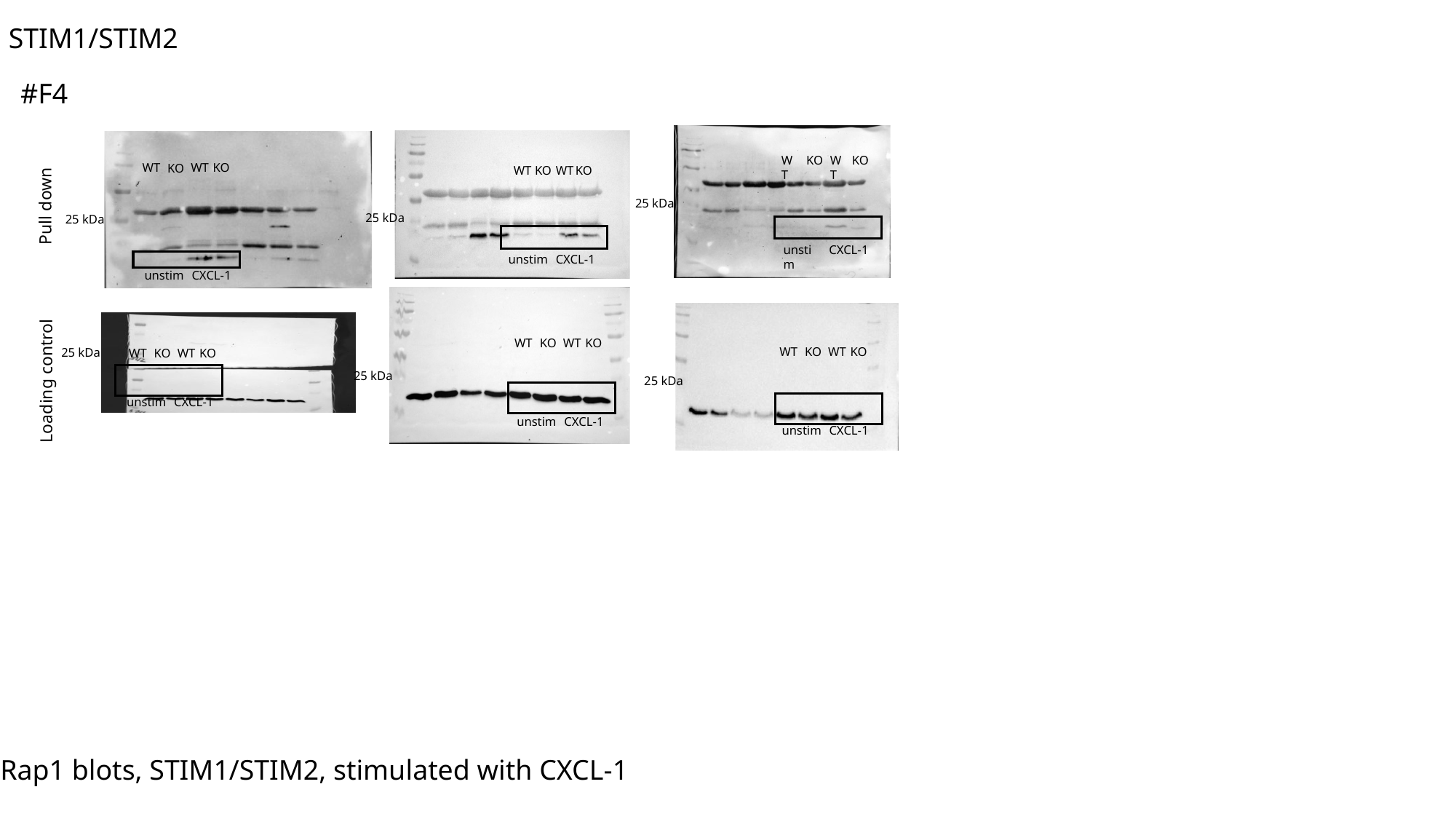

STIM1/STIM2
#F4
WT
WT
KO
KO
WT
WT
KO
KO
WT
KO
WT
KO
Pull down
25 kDa
25 kDa
25 kDa
unstim
CXCL-1
unstim
CXCL-1
unstim
CXCL-1
WT
WT
KO
KO
WT
WT
KO
KO
25 kDa
WT
WT
KO
KO
25 kDa
Loading control
25 kDa
unstim
CXCL-1
unstim
CXCL-1
unstim
CXCL-1
Rap1 blots, STIM1/STIM2, stimulated with CXCL-1

## Slide 18
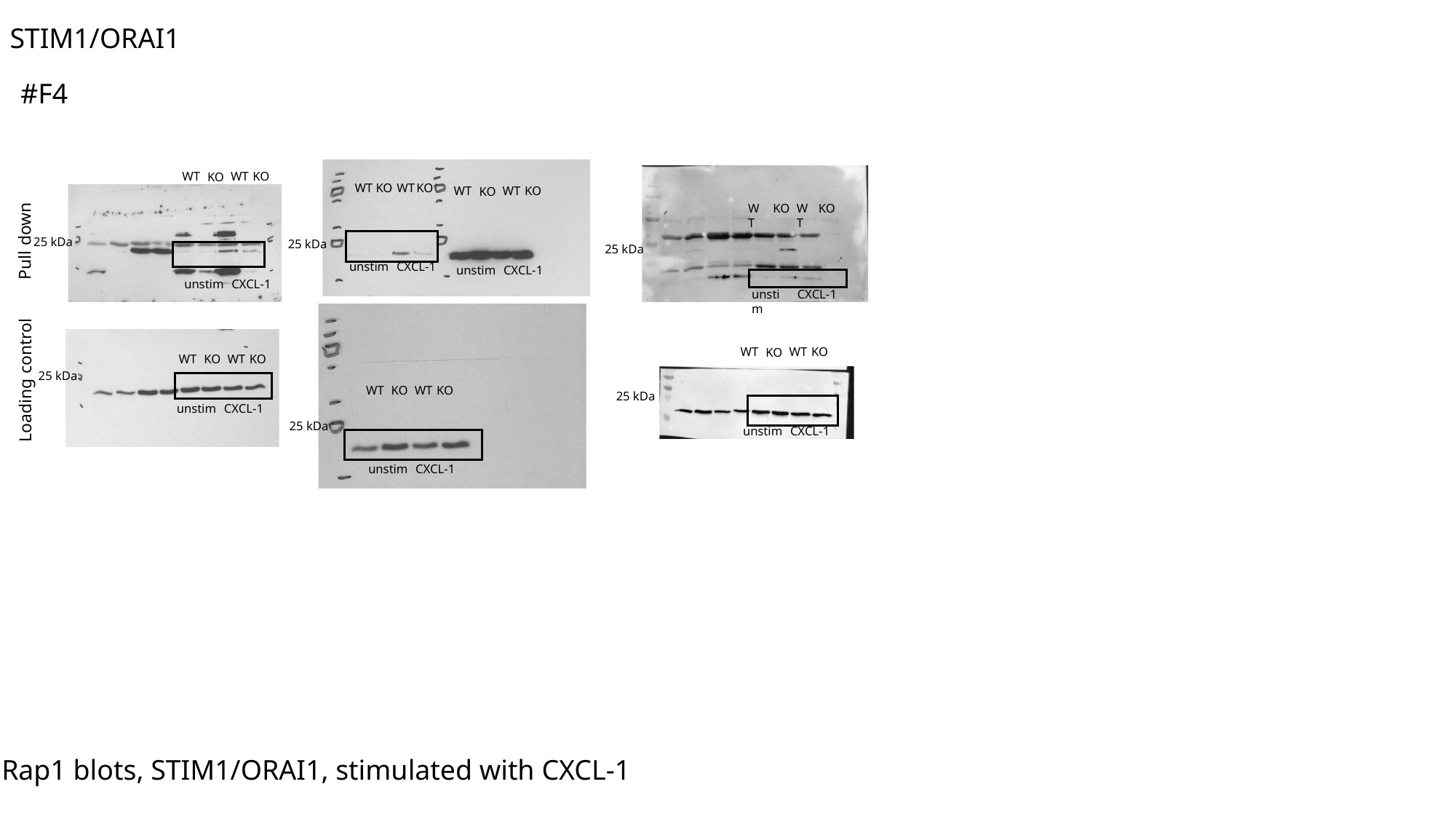

STIM1/ORAI1
#F4
WT
WT
KO
KO
WT
KO
WT
KO
WT
WT
KO
KO
WT
WT
KO
KO
Pull down
25 kDa
25 kDa
25 kDa
unstim
CXCL-1
unstim
CXCL-1
unstim
CXCL-1
unstim
CXCL-1
WT
WT
KO
KO
WT
WT
KO
KO
25 kDa
Loading control
WT
WT
KO
KO
25 kDa
unstim
CXCL-1
25 kDa
unstim
CXCL-1
unstim
CXCL-1
Rap1 blots, STIM1/ORAI1, stimulated with CXCL-1

## Slide 19
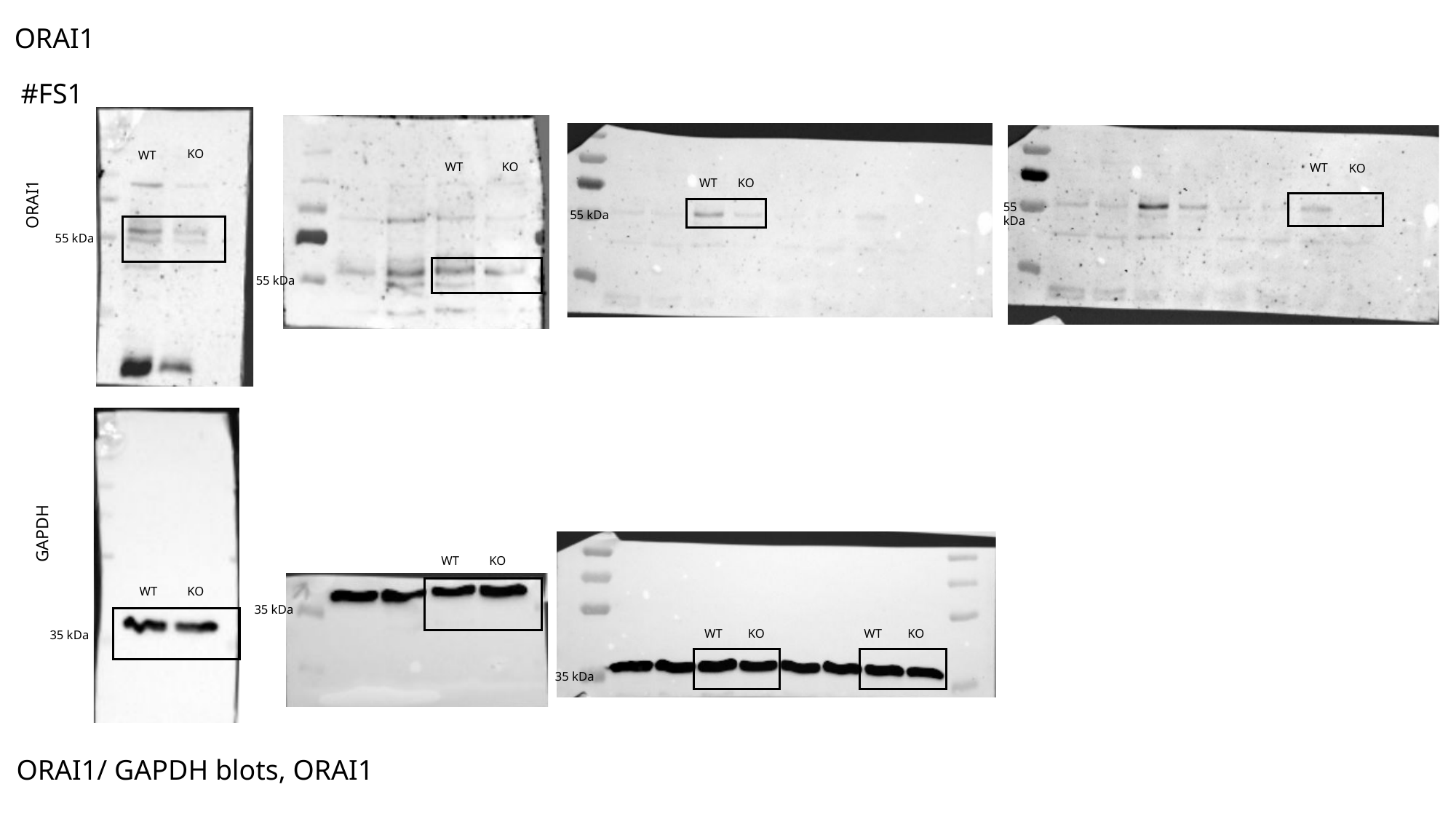

ORAI1
#FS1
KO
WT
WT
KO
WT
KO
KO
WT
ORAI1
55 kDa
55 kDa
55 kDa
55 kDa
GAPDH
WT
KO
WT
KO
35 kDa
WT
KO
WT
KO
35 kDa
35 kDa
ORAI1/ GAPDH blots, ORAI1

## Slide 20
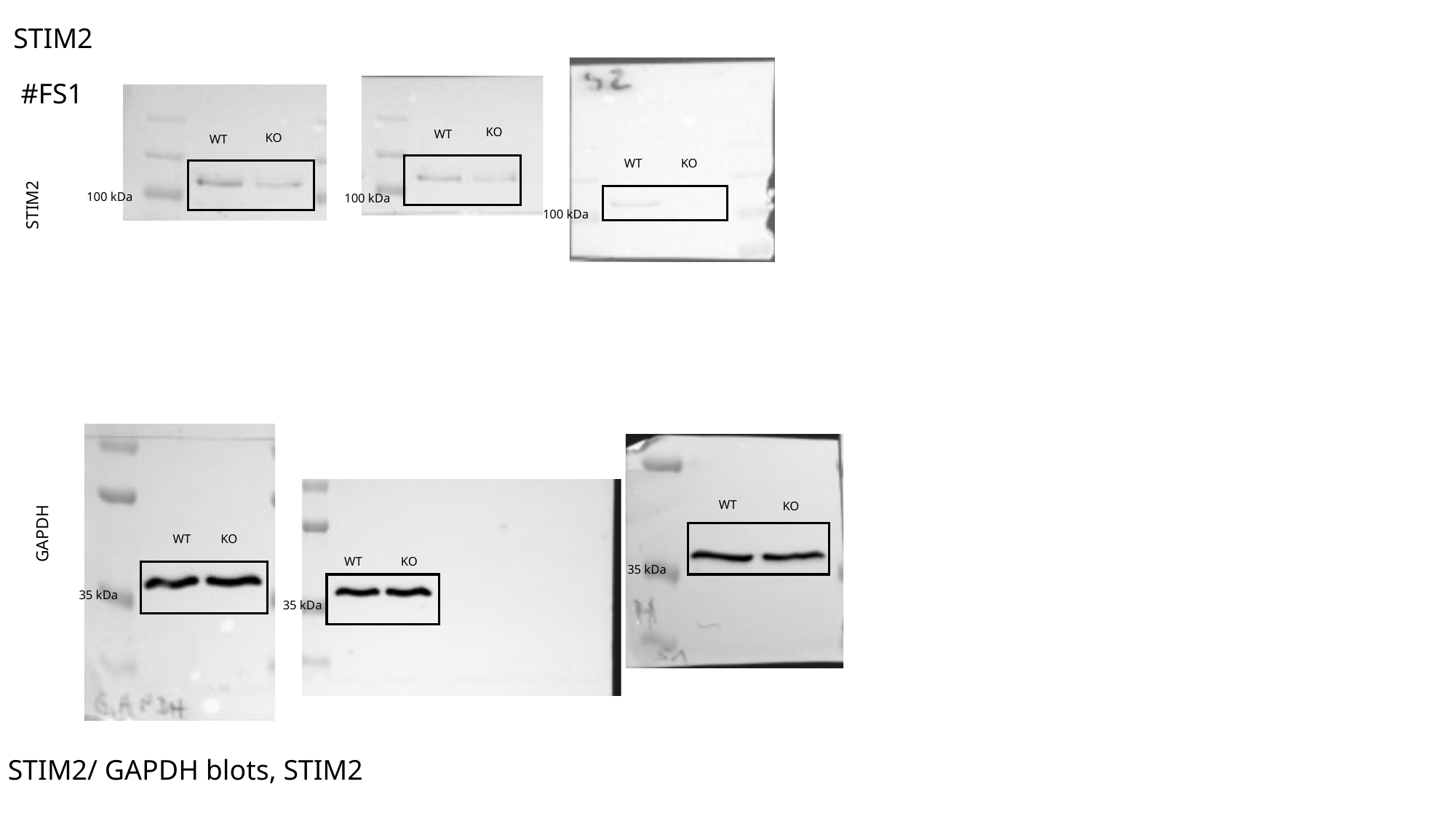

STIM2
#FS1
KO
WT
KO
WT
WT
KO
100 kDa
100 kDa
STIM2
100 kDa
WT
KO
GAPDH
WT
KO
WT
KO
35 kDa
35 kDa
35 kDa
STIM2/ GAPDH blots, STIM2

## Slide 21
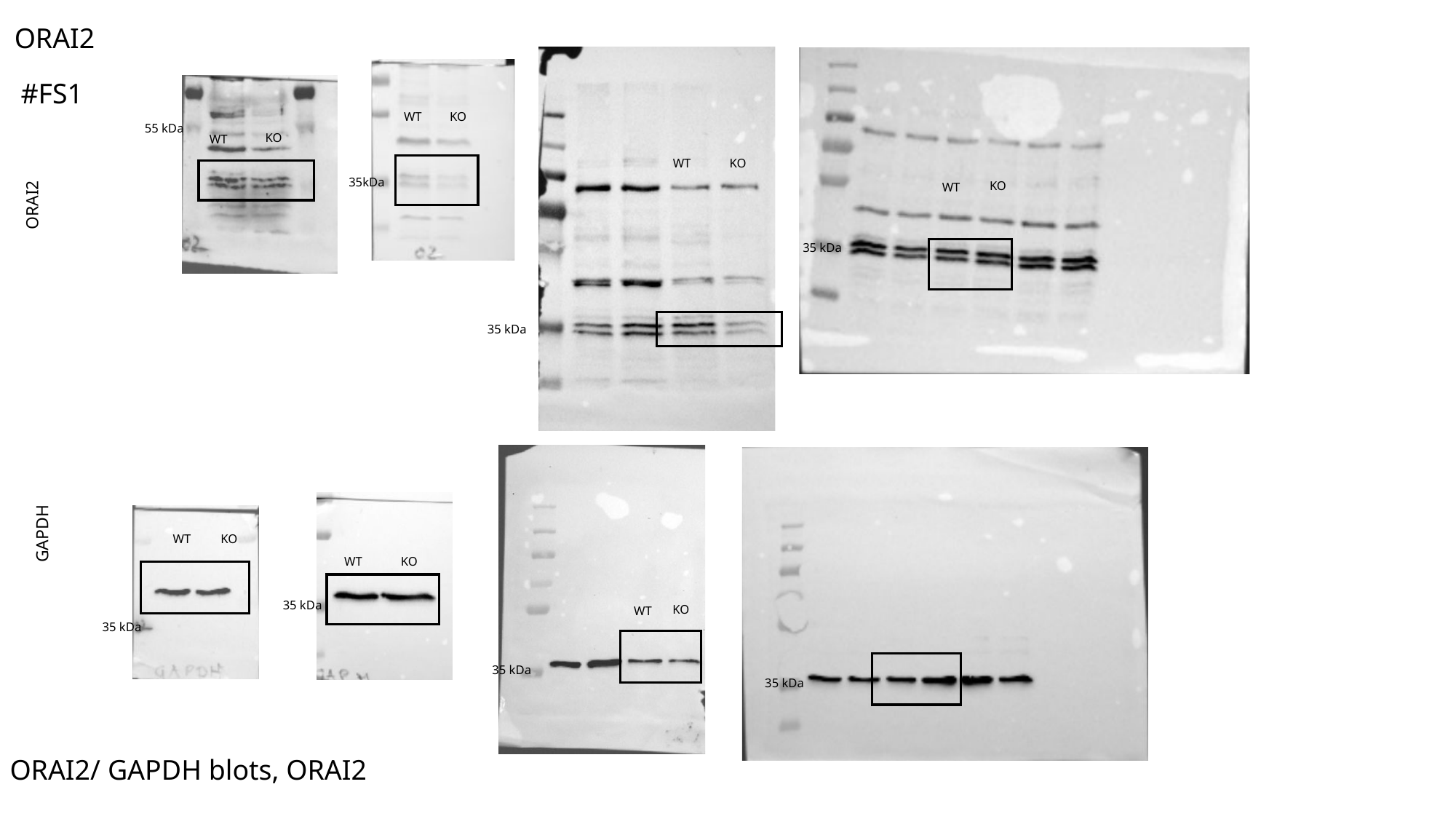

ORAI2
#FS1
WT
KO
55 kDa
KO
WT
WT
KO
35kDa
KO
WT
ORAI2
35 kDa
35 kDa
GAPDH
WT
KO
WT
KO
35 kDa
KO
WT
35 kDa
35 kDa
35 kDa
ORAI2/ GAPDH blots, ORAI2

## Slide 22
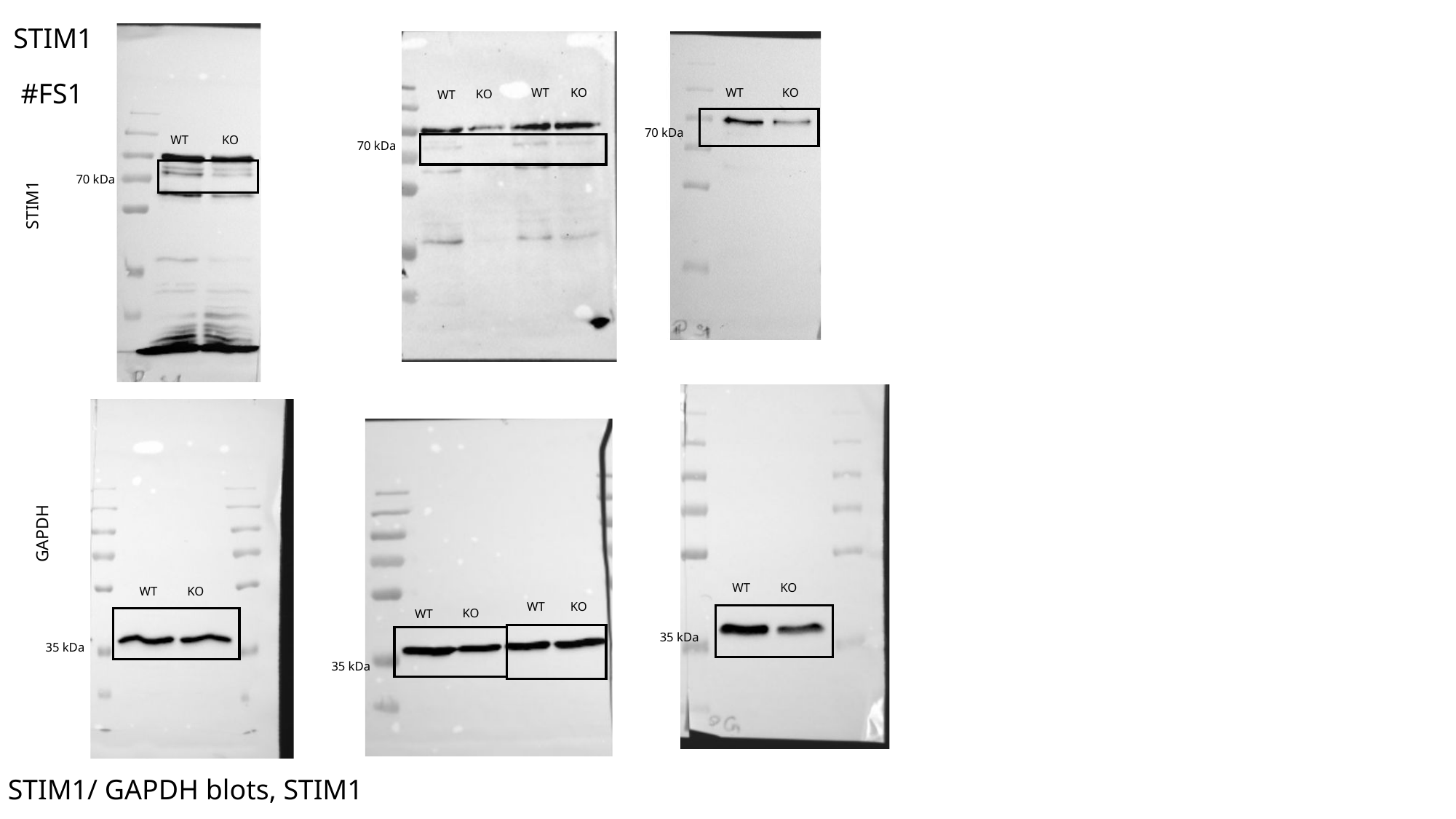

STIM1
#FS1
WT
WT
KO
KO
KO
WT
70 kDa
WT
KO
70 kDa
70 kDa
STIM1
GAPDH
WT
KO
WT
KO
WT
KO
KO
WT
35 kDa
35 kDa
35 kDa
STIM1/ GAPDH blots, STIM1

## Slide 23
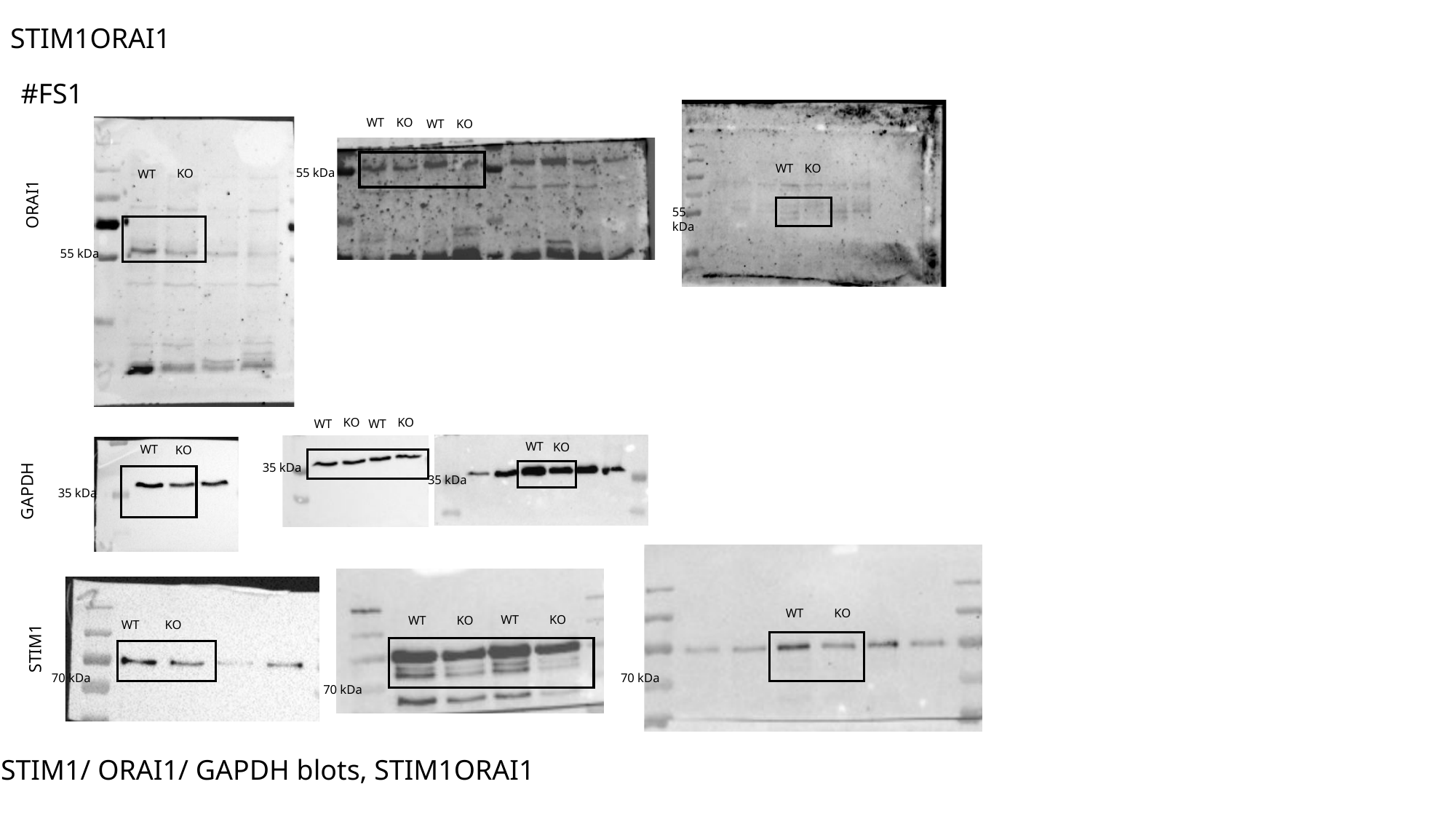

STIM1ORAI1
#FS1
WT
KO
WT
KO
WT
KO
55 kDa
KO
WT
ORAI1
55 kDa
55 kDa
KO
KO
WT
WT
WT
KO
WT
KO
35 kDa
35 kDa
GAPDH
35 kDa
WT
KO
WT
KO
WT
KO
WT
KO
STIM1
70 kDa
70 kDa
70 kDa
STIM1/ ORAI1/ GAPDH blots, STIM1ORAI1

## Slide 24
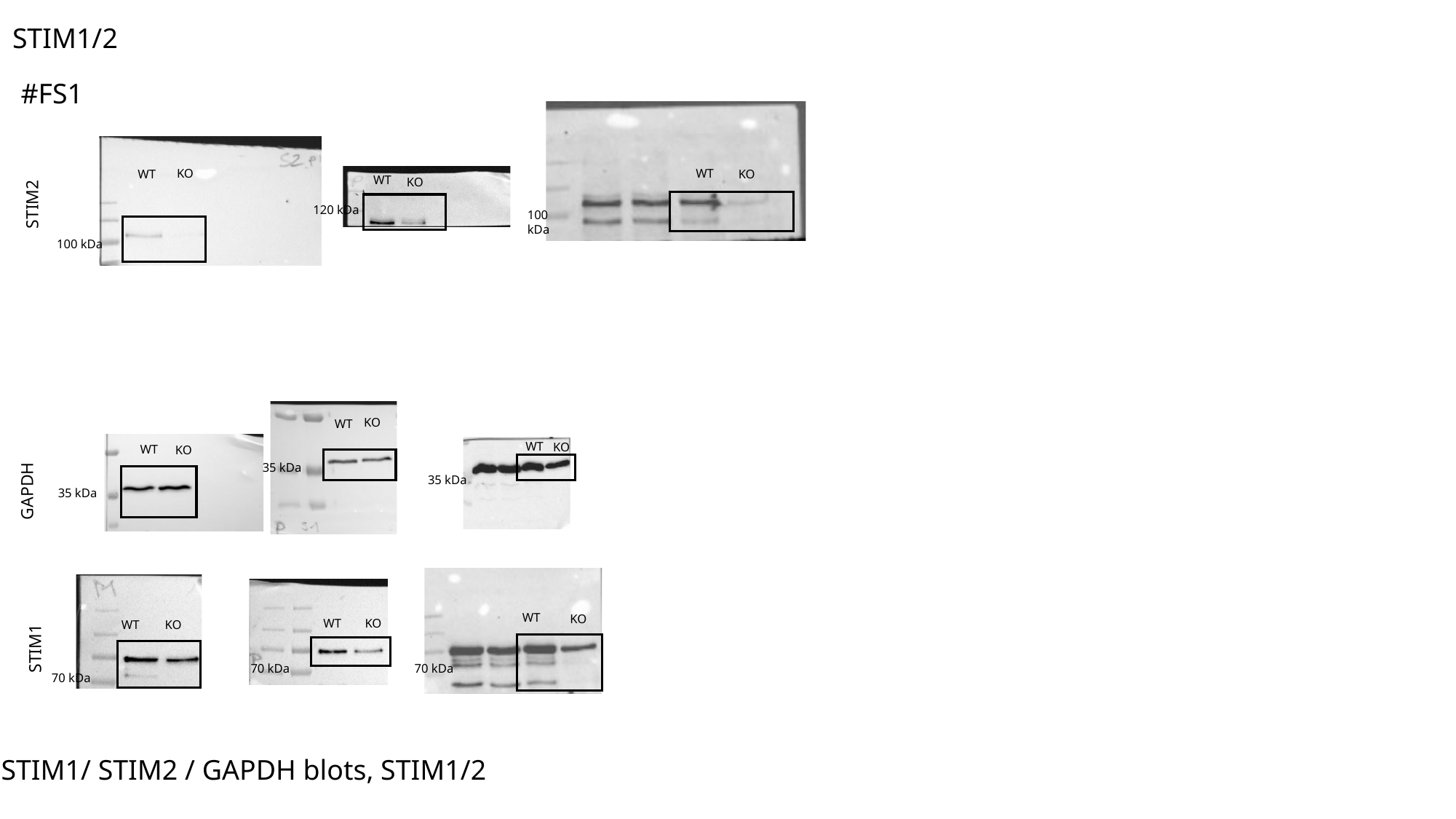

STIM1/2
#FS1
KO
WT
WT
KO
WT
KO
STIM2
120 kDa
100 kDa
100 kDa
KO
WT
WT
KO
WT
KO
35 kDa
35 kDa
GAPDH
35 kDa
WT
KO
WT
KO
WT
KO
STIM1
70 kDa
70 kDa
70 kDa
STIM1/ STIM2 / GAPDH blots, STIM1/2
